# Amplicon sequencing reveals the cryptic diversity in the dicyemid parasites of coleoid cephalopods sampled from the Atlantic and Pacific Oceans

**DOI:** 10.1007/s42995-026-00353-w

**Published:** 2026-02-17

**Authors:** Tijana Cvetković, Masoud Nazarizadeh, Tereza Koudelková, Fedor Lishchenko, Yen H. T. Dinh, Eduardo Almansa, Hannah Osland, Tomáš Scholz, Zdeněk Lajbner, Qiaz Q. H. Hua, Marie Drábková, Jan Štefka

**Affiliations:** 1 Institute of Parasitology, Biology Centre CAS, České Budějovice, 370 05 Czech Republic; 2 Department of Biological Sciences, University at Buffalo, Buffalo, NY 14260 USA; 3 Faculty of Science, University of South Bohemia, České Budějovice, 370 05 Czech Republic; 4 A.N. Severtsov Institute of Ecology and Evolution of the RAS, Moscow, 119071 Russia; 5 Coastal Branch of the Joint Vietnam—Russia Tropical Science and Technology Research Center, Nha Trang, 650000 Vietnam; 6 Centro Oceanográfico de Canarias, Instituto Español de Oceanografía (IEO), CSIC, Santa Cruz de Tenerife, 38180 Spain; 7 College of Science and Technology, Temple University, Philadelphia, PA 19122 USA; 8 Physics and Biology Unit, Okinawa Institute of Science and Technology Graduate University, Okinawa, 904-0495 Japan; 9 Environment Institute, Department of Ecology and Evolution, The University of Adelaide, Adelaide, SA 5005 Australia; 10 Faculty of Science, University of Hradec Králové, Hradec Králové, 500 03 Czech Republic

**Keywords:** Dicyemida, Cephalopods, Host–parasite assemblage, Amplicon sequencing, Biodiversity

## Abstract

Dicyemids (phylum Dicyemida), primarily found in the renal organs of coleoid cephalopods, are a unique group of morphologically simple parasites with global distribution. Here, we investigated the diversity and prevalence of dicyemid communities in a wide range of cephalopod hosts across four geographic zones (the North East Atlantic, Mediterranean Sea, China Sea in the Western North Pacific, and Australia in the South Pacific) using Illumina sequencing of the *18S rDNA* amplicons. Across 227 host samples, we identified 482 amplicon sequence variants, which clustered into 95 genetic types. The results indicated a higher number of distinct genetic types within Dicyemida than those currently identified through morphology-based taxonomy. Our finding of 46 dicyemid types in the common cuttlefish (*Sepia officinalis*) contrasts sharply with the previous records of a maximum of four species in this host. Furthermore, only a few host species exhibited a single dicyemid type, while most harbored multiple types; several types were distributed worldwide. Additionally, we identified eight new cephalopod hosts in the Pacific. Analyses of community (α) diversity suggested the unique character of certain geographical areas, such as the Bass Strait (Australia). β-diversity analyses confirmed that geographic location and host species were significant determinants of the dicyemid community composition. These results suggest that current species classifications may underestimate the true diversity of dicyemids. They emphasize the intricate interplay between geography, host specificity, and dicyemid community diversity.

**Supplementary Information:**

The online version contains supplementary material available at 10.1007/s42995-026-00353-w.

## Introduction

Delineating the genetic diversity of parasitic and mutualistic organisms helps understand host–symbiont interactions, particularly in terms of their geographic distribution or host specificity, which are key players driving their evolution and adaptability (Bouzid et al. 2008; Combes 2001; Nazarizadeh et al. 2023). Under geographic isolation, symbionts often develop distinct genetic traits by coevolving with the local host populations (Whiteman and Parker 2004). Host specificity further shapes this process, with parasites closely linked to certain hosts developing specialized genetic diversity, in contrast to those with broader host ranges who can adapt more diversely (Lajeunesse and Forbes 2002; Thompson 2005). Ultimately, the interplay between geographical and host-specific factors is crucial in shaping the genetic diversity and evolutionary trajectory of parasites, an association that is essential for formulating effective parasite management and biodiversity conservation strategies (Hudson et al. 2002).

We provide insights into the genetic diversity of a geographically widespread group of marine worms, Dicyemida, parasites of cephalopods. Dicyemida is a monophyletic yet enigmatic taxon. Historically, it was classified within the now-disputed phylum Mesozoa (which includes Orthonectida, parasites associated with benthic marine invertebrates), but their exact place in the evolutionary tree remains unresolved despite extensive research (Bleidorn 2019; Catalano 2012; Drábková et al. 2022; Dunn et al. 2014; Lapan and Morowitz 1975; Lu et al. 2017; Schiffer et al. 2018; Stunkard 1954; Suzuki et al. 2010; Telford et al. 2015; Zrzavý 2001; Zverkov et al. 2019). Compared to Orthonectida, Dicyemids represent a more specious group with just over 120 species described (Catalano 2012; Tedesco et al. 2020). They possess a simple body structure, comprising dozens of cells and lacking body cavities or differentiated organs (Furuya et al. 2003; Suzuki et al. 2010). Dicyemids are internal parasites of coleoid cephalopods, densely covering the surfaces of their renal organs and reaching densities of up to thousands of individuals per cm^3^; however, they have rarely been reported in teuthoid decapods (Drábková et al. 2021; Furuya and Tsuneki 2005; Lu et al. 2019; Suzuki et al. 2010) (Supplementary Fig. S1). Due to high density, prevalence reaching up to 100% of the host population, and apparently asymptomatic infection (Catalano et al. 2014; Finn et al. 2005; Nouvel 1947), they are sometimes suspected to be mutualistic rather than parasitic organisms.

The intimate relationship between dicyemids and their cephalopod hosts allows exploration of the complexities of coevolution and adaptation strategies in a relatively controlled and accessible setting (Lapan and Morowitz 1975). Furthermore, the diversity of dicyemid species identified in various cephalopod hosts across different geographic regions highlights the influence of environmental factors on parasitic evolution (Poulin 2007). Monitoring their diversity is crucial for understanding the ecological balance within cephalopod populations and also for gaining broader ecological insights (Johnson and Thieltges 2010). For instance, alterations in dicyemid populations could signal shifts in cephalopod health or environmental conditions, making them valuable indicators in marine ecosystems (Catalano et al. 2014; Rich et al. 2023). Thus, studying dicyemids contributes remarkably to the comprehension of parasitic relationships and their impact on broader ecological systems.

Historically, research on dicyemid species diversity has predominantly focused on their morphology, particularly the shape of the calotte (“head”), which attaches to the folds of the renal organs. Additionally, the size, number, and arrangement of peripheral cells, especially in the infusoriform larvae, serve as key markers for species identification. The cell composition of the vermiform and infusoriform stages, including specific counts and types of cells (e.g., urn, apical, and capsule cells), reflects species-specific developmental regulatory mechanisms (Furuya and Souidenne 2019). However, based solely on these morphological descriptions, a maximum of three dicyemid species have been identified within a host (Furuya 1999; Furuya and Souidenne 2019; Hochberg 1990). However, the degree to which such estimates are affected by possible intraspecific morphological plasticity remains uncertain. In contrast, recent advancements in genetics and genomics have begun to shed light on the unique life strategies of dicyemids (Drábková et al. 2021; Eshragh and Leander 2014; Suzuki et al. 2010). For example, Drábková et al. (2019) raised the possibility of the population structure of dicyemids largely mirroring the geographic distribution of their hosts. Marine species may face fewer dispersal obstacles than terrestrial ones, implying extensive distribution ranges and low interpopulation genetic variations (Palumbi 1992), with many species exhibiting marked diversification. For example, the golden cuttlefish (*Sepia esculenta*) demonstrates a broad genetic diversity (Zheng et al. 2009), attributable to biological traits and physical barriers, such as sea or ocean currents (Palumbi 1992). Thus, the degree to which the geographical distribution and local species diversity of hosts affect the species diversity of their associated dicyemids remains ambiguous.

In this research, we utilized amplicon sequencing and several species delineation methods to unravel the diverse and largely uncharted species diversity of dicyemids and their ecological interactions. We analyzed 227 cephalopod specimens representing 24 species collected from various locations around the world. We focused on several key objectives: (a) investigating the rate of prevalence of dicyemids in different hosts and across regions; (b) exploring whether multiple dicyemid species coexist in individual hosts; and (c) estimating the species and community diversities of dicyemid lineages among the sampled hosts and across regions. The results can help better gauge the probable number of existing dicyemid species and their diversity structure on a global scale.

## Materials and methods

### Sample collection and DNA extraction

**Fig. 1:**
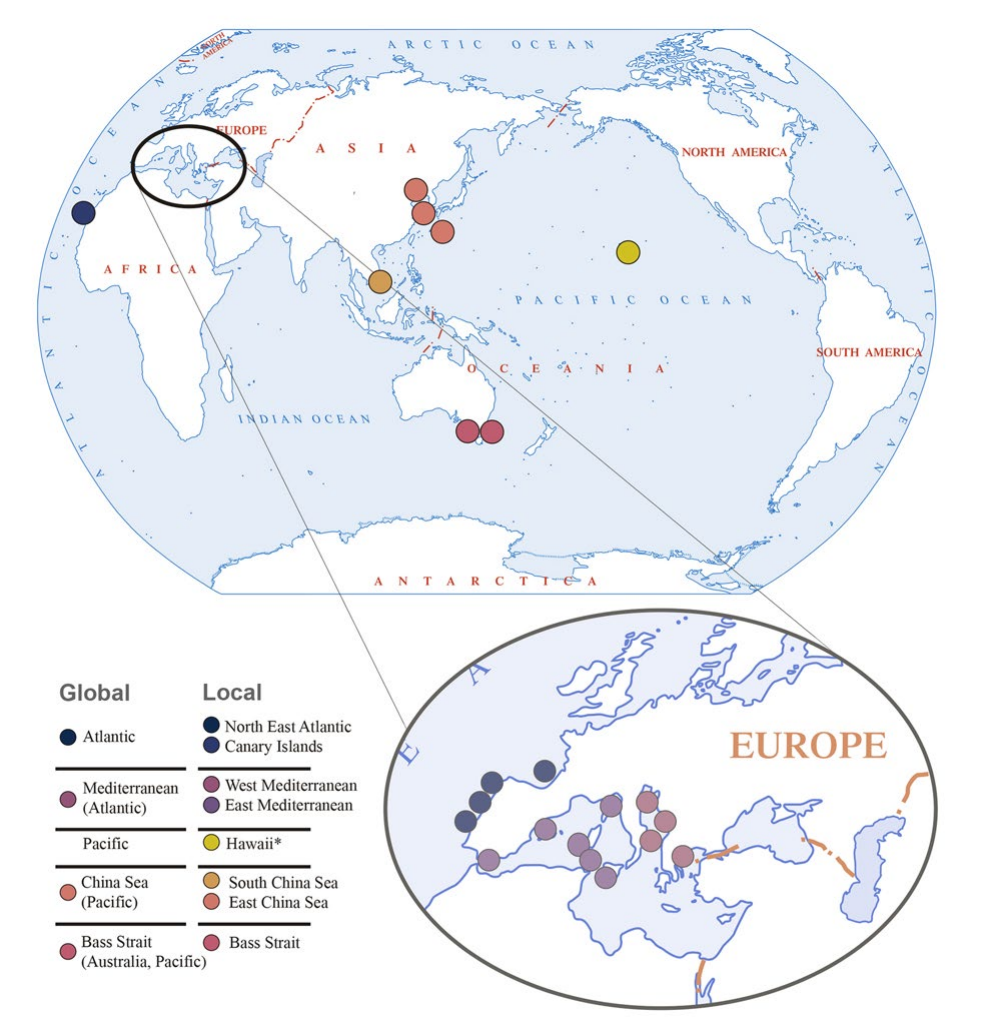
The distribution map of the sampling locations. Different colors denote global sampling areas: Atlantic—blue black, Mediterranean—light purple, China Sea—pink, Bass Strait—dark pink; and local sampling areas: North East Atlantic Ocean—blue black, Canary Islands—dark blue, West Mediterranean—dark purple, East Mediterranean—light purple, Hawaii—yellow (*no dicyemids found), South China Sea—orange, East China Sea—pink, Bass Strait—dark pink. This color scheme is used throughout the paper

We sampled 227 individual cephalopod hosts, representing 24 species across eight geographic zones: North East Atlantic, Canary Islands (Atlantic), West Mediterranean, East Mediterranean, Hawaii, South China Sea, East China Sea, and the Bass Strait (Fig. 1; Supplementary Table S1). These samples were collected between 2015 and 2021. The cephalopods were acquired either from the local fish markets or through vessel fishing. We also examined fresh cephalopod cadavers from storage facilities. We verified the exact locations of the collection points with retailers to ensure the accuracy of the geographical origins of the samples. For a detailed description of the sampling procedure, refer to Drábková et al. 2019. After dissection, a section of the renal organ and a part of an arm (a host tissue voucher) were preserved in pure ethanol (Supplementary Fig. S1). Parts of the dicyemid samples were obtained by washing the renal organs in artificial seawater (ASW; Lapan and Morowitz 1975), followed by centrifugation of the collected liquid to concentrate the dicyemids. Subsequently, DNA was extracted from both the renal organs containing the dicyemids and the arm sample using the DNeasy Blood and Tissue kit (QIAGEN, Hilden, Germany), according to the manufacturer’s instructions.

### Sequencing methods

To verify the identity of the host species, we sequenced a 700–1000 bp segment of the cytochrome c oxidase I gene (*COI*) from the arm sample. We then compared the amplicon sequence against those available in GenBank (https://www.ncbi.nlm.nih.gov/genbank/). The sequencing procedures are detailed in Supplementary Material: Part A. To investigate dicyemid diversity, we prepared PCR amplicon libraries of a segment of the *18S rDNA* gene from the renal tissue DNA, followed by sequencing using the MiSeq platform (Illumina, CA, USA). The *18S rDNA* marker demonstrated sufficient variability in dicyemids during preliminary analyses. The primers successfully amplified the targeted region across diverse samples compared to the primers for the *COI* marker, which were too specific to apply to worldwide dicyemid samples (Drabková, pers. obs.). We employed a dual indexing approach for multiplexing samples in one sequencing run (see Supplementary Material: Part A for details), which significantly reduced costs. Bioanalyzer (Agilent Technologies Inc., CA, USA) was run to check for primer dimers, the optimal molarity of the primers, and other potential drawbacks. By utilizing heterogeneity primers, a possible sequencing failure due to a low PhiX spike-in was avoided (Chaudhry et al. 2019; Hammoud et al. 2021). As a final step, after PCR, amplicons from 353 selected samples, including duplicates and 227 unique samples, were sent for sequencing. For details concerning sample processing, refer to Supplementary Material: Part A.

### Bioinformatic analyses

FastQC v0.11.5 (Andrews 2010) and MultiQC v1.12 (Ewels et al. 2016) were used to evaluate the quality of the raw reads. Primers and adapters were excluded from the raw sequences, and low-quality sequences were removed utilizing the MetReTrim v0.1 (Sharda 2020). The reads were analyzed with the QIIME2 pipeline (Bolyen et al. 2019) using DADA2 (Callahan et al. 2016) to obtain the amplicon sequence variants (ASVs), form sequencing error profiles, and remove chimeras, with preset trimming parameters (–p-trunc-len-f = 230 and –p-trunc-len-r = 220). We used the “just concatenated” option of the DADA2 plugin to merge the non-overlapping forward and reverse reads. To construct a precise taxonomic assignment file, we obtained the latest *18S rDNA* reference sequence from the SILVA database v132 (https://www.arb-silva.de/documentation/release-132/) and manually added all the dicyemid sequences available in NCBI (https://www.ncbi.nlm.nih.gov/) to the database. The taxonomy of the ASVs was assigned using a feature-classifier plugin that was trained using 99% clustered *18S rDNA* references. The prevalence of dicyemids based on their geography and cephalopod hosts, with a 95% confidence interval, was estimated using the function “epi.conf” included in the epiR R package (Stevenson et al. 2015) for the statistical package Rv3.0 (R Core Team 2021). Then, the taxonomic ranks of the dicyemid species were curated by manual inspection. To evaluate the completeness of sampling, we generated a phylogenetic alpha rarefaction curve using QIIME2. Next, all sequences were aligned utilizing the integrated MAFFT aligner (https://mafft.cbrc.jp/alignment/server/index.html) and the rooted and unrooted 18S phylogenetic trees were constructed employing the FastTree algorithm (Price et al. 2009) within the QIIME 2 phylogenetic module.

### Phylogenetic tree analyses and species delimitation

All ASVs were aligned using the ClustalW algorithm in MEGA v6 (Tamura et al. 2013), generating a 512-bp-long data matrix. To determine whether these regions were located within the conserved or variable regions of the *18S rDNA* gene, we aligned them with 15 *18S rDNA* sequences available in GenBank and estimated the number of parsimony-informative and singleton sites using DnaSP v6 (Rozas et al. 2017). The results confirmed that this region was particularly variable, with 88 parsimony-informative and 55 singleton sites, excluding gaps and missing data. The sequence alignment is available as Supplementary Material 2. Furthermore, we tested for substitution saturation using the two-tailed *t* test method of Xia et al. (2003) implemented in DAMBE v7.2.25 (Xia 2018). The results confirmed that the observed index of substitution saturation (Iss) was significantly lower (*P* < 0.05) than the critical index (Iss.c), indicating little saturation of our data. Thus, the sequences were deemed suitable for reliable phylogenetic analysis.

We then employed PartitionFinder v2.3 (Lanfear et al. 2017) to identify the best model for DNA sequence evolution. The results indicated the GTR + I + G model as the most suitable. Based on this model, we constructed a phylogenetic tree of the ASVs employing BEAST v2.5.2 (Bouckaert et al. 2019). The parameters for this analysis included a Markov Chain Monte Carlo (MCMC) chain set to run for 200,000,000 generations, with trees sampled every 10,000 generations. Each run was assessed for convergence and effective sample size using Tracer v1.7 (Rambaut et al. 2018). We set a posterior probability limit of 0.5; the maximum clade credibility tree was generated utilizing the TreeAnnotator program, a component of the BEAST package (https://www.beast2.org/treeannotator/). The phylogenetic tree incorporated the *18S rDNA* dicyemid sequences accessed from GenBank (Supplementary Table S2) along with the ASVs detected, using lophotrochozoan and bacterial sequences as outgroups.

Given the limitations of current databases, particularly the low number of *18S rDNA* sequences available to accurately identify dicyemid species, we conducted a series of single-locus-based species delimitation analyses to explore potential species boundaries and evolutionary lineages within the dicyemid ASVs. This study analyzed a 512-bp fragment of the *18S rDNA* gene, which is more conserved than mitochondrial markers, such as *COI*, and therefore less suited for resolving fine-scale species boundaries (Ogedengbe et al. 2011; Tang et al. 2012). On the contrary, the *COI* for dicyemids is underrepresented in the databases, and it failed to amplify the DNA from a wide variety of samples in our trials. To avoid misinterpreting the results of our *18S rDNA*-based species delimitation analyses, we refrained from assigning species-level designations to the genetic lineages identified. Instead, we adopted the term “genetic type” to represent ASVs that were consistently identified as distinct evolutionary lineages across multiple species delimitation methods.

Specifically, we employed the General Mixed Yule Coalescent (GMYC; Pons et al. 2006), Bayesian GMYC (bGMYC; Reid and Carstens 2012), and Bayesian Poisson Tree Processes (bPTP; Zhang et al. 2013) methods. The GMYC analysis was implemented using the “splits” package (Ezard et al. 2009) in R, applying a threshold of 0.05 for significant interspecific differentiation. Subsequently, the bGMYC method was applied by utilizing the “bGMYC” package (Reid et al. 2013) in R (R Core Team 2021; RStudio Team 2020), with (MCMC) simulations run for 1,000,000 iterations to ensure thorough sampling of the parameter space. The bPTP method was executed using the “bPTP.py” script (Zhang et al. 2013) in Python 3, with the default settings adjusted for our analysis. To prevent the over-splitting of dicyemid types, we grouped the ASVs into dicyemid types that were consistently recognized as distinct species by all three approaches.

### Alpha and beta diversity in dicyemids

To estimate the structuring of diversity in dicyemid communities based on geography and host species, we calculated the α- and β-diversities, representing the mean local species diversity and the differentiation among local sites (or hosts), respectively (Whittaker 1960). We calculated the α- and β-diversities at two geographic scales. Initially, we categorized all host populations on a broad geographic scale within the marine environment, grouping them into four regions—the Canary Islands and Northeast Atlantic, the Mediterranean (Atlantic), the China Sea and Hawaii, and the Bass Strait—and compared their α- and β-diversities. Subsequently, we focused on a finer scale, concentrating on the more extensively sampled dicyemid populations, specifically within the Atlantic (Canary Islands and Northeast Atlantic) and two Mediterranean regions.

The taxonomic assignment file was revised for unidentified ASVs based on the findings from the species delimitation analyses. We excluded ASVs from single-infected hosts for the downstream analysis, because calculating these diversity metrics requires at least two infected samples from each group. Several α-diversity indices were calculated: Faith’s phylogenetic diversity, ACE, Shannon diversity, Shannon evenness, Simpson diversity, InvSimpson diversity, and Simpson evenness. For β-diversity assessment, we employed indices such as Bray–Curtis and weighted UniFrac distances, which were visualized through principal coordinate analysis (PCoA) plots produced using the MOCHI web application (https://mochi.life.nctu.edu.tw/; Zheng et al. 2022). We applied the Kruskal–Wallis and post hoc Dunn tests to analyze the marked differences in α-diversity between the groups. Additionally, we estimated β-diversity using Bray–Curtis distances and conducted a permutational multivariate analysis of variance (PERMANOVA; Anderson 2017) and analysis of similarities (ANOSIM; Clarke 1993) to determine the significant variations in dicyemid diversity across different hosts and localities. The results of PERMANOVA and ANOSIM provide insights into the variations and differences in community composition. In PERMANOVA, the *R*^2^ value, or the coefficient of determination, indicates the proportion of variation explained by the factor being studied (geography and host specificity). An *R*^2^ value close to 1 signifies that a high proportion of the variation can be explained by the factor, indicating a robust influence. Conversely, an *R*^2^ value close to 0 indicates that the factor minimally explains the variation, suggesting a weak influence. In ANOSIM, the *R* value ranges from − 1 to 1, where values closer to 1 indicate a greater degree of separation between groups, signifying distinct variations in community composition. Values closer to 0 indicate marginal or no separation, suggesting that the intergroup variations are minimal. Thus, high *R*^2^ and *R* values point to strong and significant impacts, while low values suggest weak or negligible effects of the factors studied (Anderson and Walsh 2013; Clarke 1993).

## Results

### Dicyemid *18S rDNA* amplicon sequencing

Sequencing produced a total of 5,148,232 raw reads (1,385,654 unique ones). After demultiplexing, between 211 and 40,692 reads per sample were obtained (mean = 7292, median = 4725). Overall, of the 353 samples sequenced (including PCR duplicates and/or separate isolates), 227 individual host samples were processed in the following analyses (Supplementary Table S1). For a detailed output containing the denoising statistics and the number of reads that passed through the whole pipeline, see Supplementary Table S3. QIIME analysis assigned reads to a total of 3855 ASVs. Due to nontargeted polymerase activity, possible sequencing errors, and contamination during lab work, the assigned taxonomic groups were manually checked for illogical hits. Next, 482 ASVs belonging to Dicyemida (out of 3855) were used for further analyses. The rest represented mostly cephalopod hosts, fungi, chromidinids, sequences of bacterial origin, and other nontarget DNA present within the renal organs. A few samples revealed possible contaminant ASVs, which could be the result of nonspecific primer annealing, cross-talk (index hopping during sequencing; MacConaill et al. 2018), or contamination during laboratory work. These contaminant ASVs were also disregarded during further analysis.

### Geographic and host-specific variability in dicyemid prevalence

Overall, 172 out of the 227 unique host samples across 19 different cephalopod species and various geographic regions showed the presence of dicyemids (Table 1). The overall prevalence reached 75%. The highest prevalence rates were found in the North East Atlantic and the Mediterranean Sea, with rates of 100% (confidence interval [CI] (92%–100%) and 91% (CI 81.52%–96.64%), respectively. In both areas, all host species routinely exhibited prevalence rates close to 100%, indicating that almost all the sampled individuals were infected (i.e., *Octopus vulgaris*, *Sepia officinalis*, *Eledone* spp., and *Callistoctopus macropus*). The Bass Strait (Australia, Pacific Ocean) also revealed a relatively high prevalence, with 79% of hosts infected (CI 65%–89%), although *Octopus pallidus* was the only species examined. In contrast, the China Sea demonstrated a moderately low prevalence, with 38% of the samples infected (CI 26%–51.78%). Although a wide range of host species was examined in that area, only a few specimens for each were available. In some instances, < 5 samples were examined. Consequently, we did not report the prevalence of such species due to the limited scientific value of the data. We obtained single samples each of *Cistopus taiwanicus*, *Amphioctopus aegina*, *A*. *ovolum*, *Sepia lycidas*, and *Nototodarus hawaiiensis* from the Pacific Ocean, all of which tested positive for dicyemids. Conversely, the single samples of *Stenoteuthis oulaniensis* and *Sepia recurvirostra* did not show the presence of any dicyemid genetic material. To our knowledge, the following species have been reported to host dicyemids for the first time: *Amphioctopus aegina*, *A*. *marginatus*, *A*. *ovolum*, *Cistopus taiwanicus*, *Octopus incella*, *O*. *laqueus*, *O*. *variabilis*, *Uroteuthis chinensis*, and *Nototodarus hawaiiensis*.

**Table 1:** Occurrence and prevalence of dicyemids in cephalopod hosts across various geographic regions, summarizing the number of infected individuals (*I*) and the total number of hosts surveyed (*N*). Prevalence (rate of infection, *P*) and a 95% confidence interval (CI) were reported for cephalopod hosts and marine environments where at least five specimens per category were available

Geographic areas	Total P (CI)	Host	*N*	*I*	*P* (CI)
Canary Islands and NE Atlantic	100% (92–100)	*Eledone cirrhosa*	4	4	–
		*Octopus vulgaris*	24	24	100% (85.7–100)
		*Sepia officinalis*	22	22	100% (84.6–100)
Mediterranean (Atlantic)	91% (81.5–96.6)	*Callistoctopus macropus*	5	5	100% (47.9–100)
		*Eledone cirrhosa*	4	4	–
		*Eledone moschata*	25	21	84% (63.9–95.5)
		*Octopus vulgaris*	4	3	–
		*Sepia elegans*	4	4	–
		*Sepia officinalis*	25	24	96% (79.6–99.9)
China Sea (Pacific ocean)	38.3% (26–51.8)	*Abdopus aculeatus*	9	1	11.1% (0.3–48.3)
		*Amphioctopus aegina*	1	1	–
		*Amphioctopus marginatus*	2	2	–
		*Amphioctopus ovolum*	1	1	–
		*Cistopus taiwanicus*	1	1	–
		*Metasepia tullbergi*	6	0	0%
		*Nototodarus hawaiiensis*	1	1	–
		*Octopus incella*	2	1	–
		*Octopus laqueus*	7	1	14.3% (0.4–57.8)
		*Octopus variabilis*	12	11	91.7% (61.5–99.8)
		*Sepia lycidas*	1	1	–
		*Sepia recurvirostra*	1	0	–
		*Sepioteuthis lessoniana*	11	1	9.1% (0.2–41.3)
		*Stenoteuthis oualaniensis*	1	0	–
		*Uroteuthis duvauceli*	2	0	–
		*Uroteuthis chinensis*	2	1	–
Hawaii (Pacific ocean)	–	*Euprymna scolopes*	2	0	–
Bass strait (Australia, Pacific)	79.2% (65–89)	*Octopus pallidus*	48	38	79.2% (65–89.5)

### Phylogenetic relationships and species delimitation of the ASVs

**Fig. 2:**
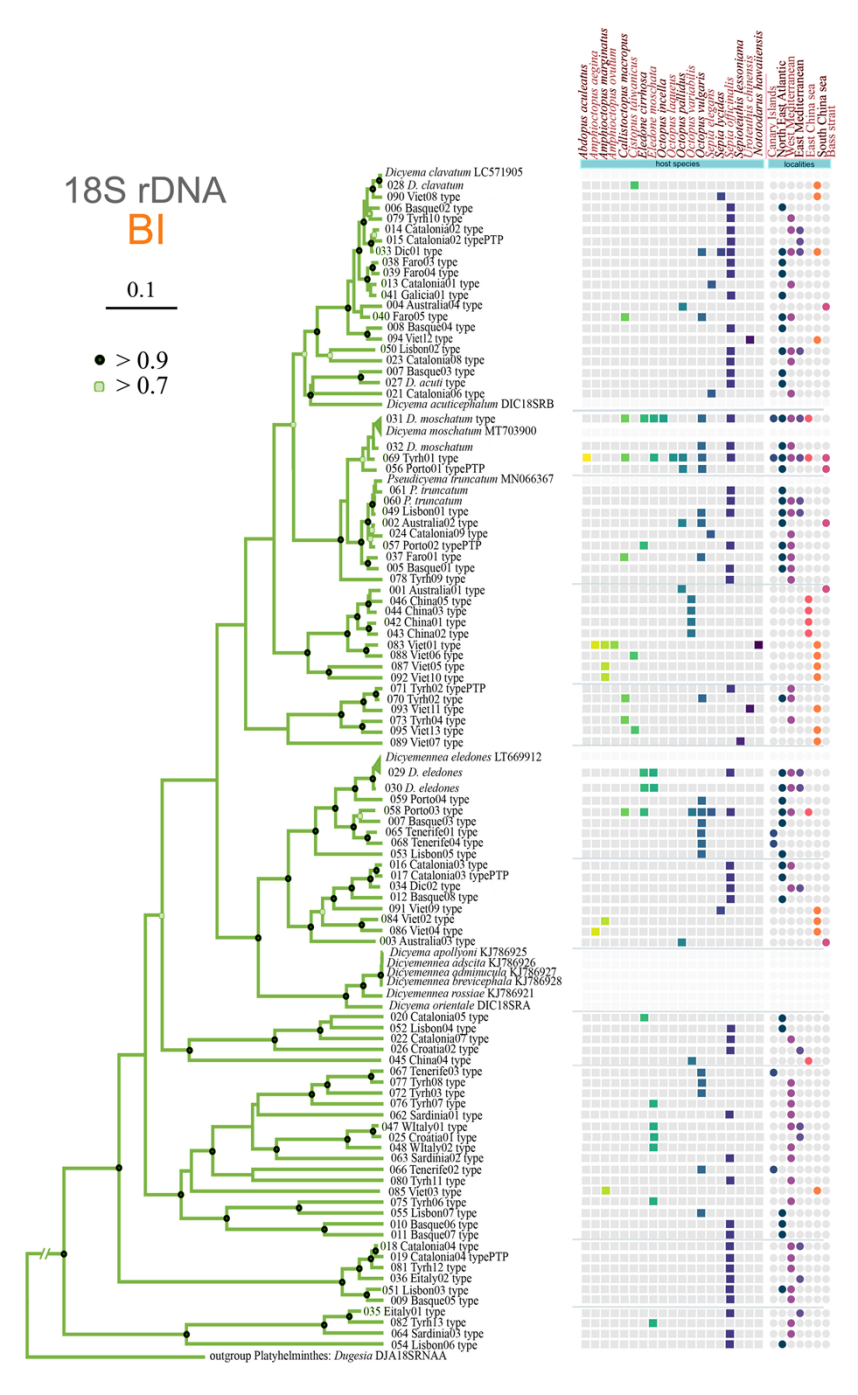
Graphical summary of the dicyemid genetic diversity and phylogenetic tree of the genetic types described by species delimitation methods in the context of the host species and the geographical origin of a sample. Each point represents the presence of a dicyemid type, which is color-coded and organized by host species and location

The results of the species delimitation analyses were not congruent, with estimates ranging from 98 to 104 species or types (GMYC and bPTP). However, all three methods—GMYC, bGMYC, and bPTP— consistently identified 95 types among the 482 ASVs. Notably, four morphologically distinct species, *Dicyema apollyoni*, *Dicyemennea adscita*, *Dicyemennea adminicula*, and *Dicyemennea brevicephala* (GenBank accessions: KJ786925, KJ786926, KJ786927, and KJ786928, respectively), were consistently clustered into one genetic type by all methods (the bright yellow cluster in the lower half of Supplementary Fig. S2). The Bayesian phylogenetic tree constructed employing the *18S rDNA* amplicons of dicyemids shows the genetic dicyemid types in the context of the available reference sequences (Fig. 2). The branching pattern of the tree suggests that a majority of the nodes have a posterior probability > 0.9, indicating relatively robust support for the phylogenetic relationships depicted. *Dicyema* and *Dicyemennea* failed to form monophyletic groups. A plot on the right side of the tree visualizes the occurrence of various dicyemid types concerning their hosts and geographic distribution.

In total, we identified 95 dicyemid genetic types; several types may be present in a variety of hosts across different geographic regions. Most of these types (70/95) were unique for one host, and 25 were shared between ≥ 2 hosts. Additionally, three types were shared by six hosts and were worldwide in distribution (031_D_moschatum, 058_Porto03, and 069_Tyrh01). Overall, *Sepia officinalis* exhibited the maximum number of dicyemid types (46), followed by *Octopus vulgaris* (20) and *Eledone moschata* (10). Some hosts contained only shared genetic types (*Abdopus aculeatus*, *Amphioctopus ovolum*, *Nototodarus hawaiiensis*, *Octopus incella*, and *Octopus laqueus*). In contrast, others only demonstrated unique genetic types (*Cistopus taiwanicus*, *Sepioteuthis lessoniana*, and *Uroteuthis chinensis*). Most of the hosts examined contained a mixture of unique and shared types (e.g., *Amphiocotpus marginatus*, *Octopus variabilis*, *Sepia elegans*, and *Sepia lycidas*). Notably, 083_Viet01 was common to several different hosts within the same locality. In contrast, the cephalopods *Euprymna scolopes*, *Metasepia tullbergi*, *Stenoteuthis oulaniensis*, *Sepia recurvirostra*, and *Uroteuthis duvauceli* hosted no dicyemids (Supplementary Table S4; Fig. 2).

The highest number of dicyemid genetic types was found in the West Mediterranean (43), followed by the North East Atlantic and East Mediterranean. The number in other areas was comparatively lower, with Hawaii showing no presence of dicyemids. However, this result may be due to the sampling strategies employed (see Discussion). Most dicyemid genetic types were found in only one locality (71); several were shared between ≥ 2, usually geographically close localities (i.e., the West and East Mediterranean). However, six dicyemid types, 002_Australia02, 031_D_moschatum, 033_Dic01, 056_Port01, 058_Port03, and 069_Tyrh01, showed a broad geographical distribution (Supplementary Table S4). At the level of individual hosts, we identified between one and eight dicyemid types within a single host. The average number of dicyemid types in one host individual was 2.3, which differed with host species. *Octopus pallidus* individuals generally hosted only one dicyemid type. In contrast, *Sepia officinalis* individuals usually hosted > 3 types, with some samples containing up to eight dicyemid types, the maximum recorded among the examined samples (*Octopus pallidus* average: 1.1, *Sepia officinalis*: 3.4; Supplementary Table S5).

### Alpha and beta diversity

#### Global scale

**Fig. 3:**
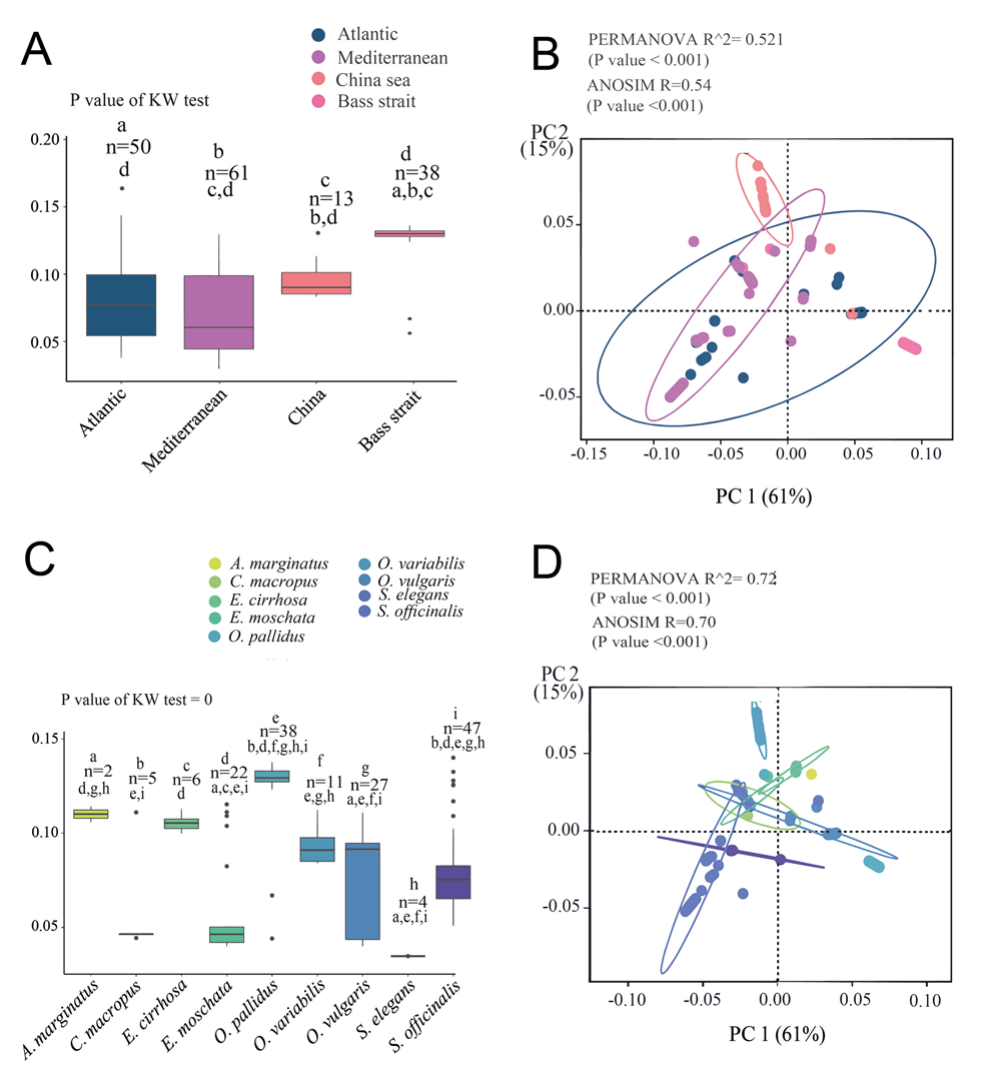
Analysis of α- and β- (weighted UniFrac Principal Coordinates Analysis [PCoA]) diversities of dicyemid communities using Faith’s Phylogenetic Diversity (PD). **A** Faith’s PD measured across four major regions: Atlantic, Mediterranean, China Sea, and Bass Strait, with each boxplot displaying the distribution of biodiversity values and annotations indicating statistically significant differences. **B** PCoA based on the PERMANOVA and ANOSIM results, illustrating clustering by geographic regions and variance explained by the principal components. **C** Faith’s PD across nine different cephalopod species, highlighting species-specific biodiversity levels. **D** PCoA demonstrating species-specific clustering and the relationships between cephalopod biodiversities across various locations. Letters below the sample numbers represent groups different from the ones selected in the Kruskal–Wallis test

The α- and β-diversity metrics were calculated for 162 out of 172 positive samples. However, ten samples were excluded, because these metrics require at least two infected samples from each group. The results of the α-diversity analysis, based on Faith’s phylogenetic diversity (PD), revealed that dicyemid diversity in the Bass Strait was significantly greater than that in other marine environments (Fig. 3A; Atlantic, China Sea, and Mediterranean; *P* < 0.05). Similarly, the PCoA plot for β-diversity demonstrated that PCO1 and PCO2 could explain 61% and 15% of the total variance, respectively (Fig. 3B). These results indicate that the Bass Strait diverged from other localities along the PCoA1 axis and the China Sea along the PCoA2 axis. Moreover, dicyemids revealed marked differences in α-diversity across different cephalopod hosts (*P* < 0.05). The α-diversity of *A*. *marginatus*, *E*. *cirrhosa*, *O*. *pallidus*, and *O*. *variabilis* was significantly higher than *C*. *macropus*, *E*. *moschata*, and *S*. *elegans* (Fig. 3C). However, *O*. *vulgaris* and *S*. *officinalis* showed no marked variations compared with other cephalopod hosts. Additionally, the β-diversity analysis revealed a clear structural pattern in dicyemid diversity across *A*. *marginatus*, *E*. *cirrhosa*, *O*. *pallidus*, and *O*. *variabilis* (Fig. 3D).

The results of the rarefaction analysis suggested that our estimates of the diversity of dicyemid types across hosts were not affected by the sampling depth. Faith’s PD plots for each host plateaued at ~ 200 read depth, indicating that additional sequencing depth did not significantly enhance the observed diversity, whereas a majority of the samples showed > 200 reads (Supplementary Fig. S3). The α-diversity, measured through a variety of indices, was fairly consistent with Faith’s PD, revealing marked variations among the Atlantic, Bass Strait, China Sea, and Mediterranean regions (*P* < 0.05; Supplementary Fig. S4). The dicyemid community from the Bass Strait demonstrated the greatest α-diversity compared to the other three regions, whereas the Atlantic and Mediterranean regions had relatively similar α-diversity levels. In addition, the Bass Strait appeared to have a more balanced ecosystem with greater evenness, as indicated by its higher Shannon and Simpson evenness index values (Supplementary Fig. S4).

Moreover, the α-diversity of dicyemids revealed that certain cephalopod species harbor remarkably richer and more diverse dicyemid communities than others. For instance, *E*. *moschata* and *O*. *pallidus* are characterized by high species richness, as indicated by various α-diversity indices, suggesting a complex ecosystem with a multitude of dicyemid species. In contrast, species such as *A*. *marginatus*, *C*. *macropus*, and *O*. *variabilis* demonstrated a relatively low α-diversity. However, the low sample size for *A*. *marginatus* may limit the reliability of its diversity estimated in this study. Additionally, *Sepia officinalis* exhibited high species richness but a wide range of α-diversity, which was insignificantly different compared to other cephalopod hosts (Supplementary Fig. S5). The results from the Bray–Curtis dissimilarity analysis aligned with the β-diversity findings from Faith’s PD, revealing a significant divergence in dicyemid diversity within the Bass Strait in *O*. *pallidus* hosts compared with other groups. This divergence was demonstrated along the PCoA1 axis, which explained 14% of the variance. The statistical significance of these patterns was confirmed by the PERMANOVA and ANOSIM tests, with PERMANOVA indicating a moderate-effect size (*R*^2^ = 0.23) and ANOSIM confirming a robust dissimilarity (*R* = 0.37), both significant at *P* < 0.001 (Supplementary Fig. S6).

**Fig. 4:**
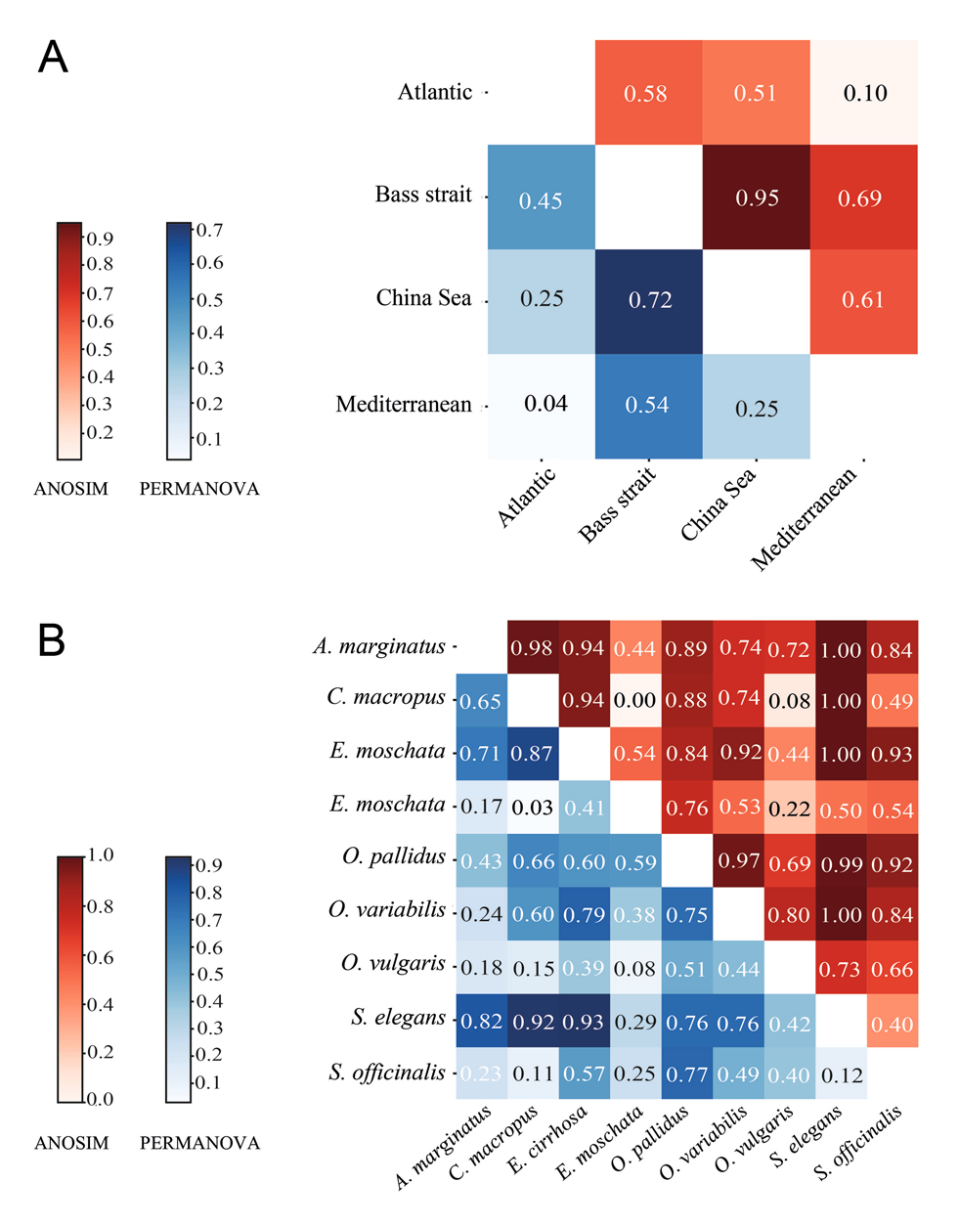
Pairwise comparisons of β-diversity using weighted UniFrac distances for Analysis of Similarities (ANOSIM R, upper) and Permutational Multivariate Analysis of Variance (PERMANOVA *R*^2^, lower) across different localities and various hosts. **A** Pairwise comparisons among four marine regions: Atlantic, Bass Strait, China Sea, and Mediterranean. **B** Pairwise comparisons among various cephalopod hosts, including the coconut octopus (*Amphioctopus marginatus*), Atlantic white-spotted octopus (*Callistoctopus macropus*), curled octopus (*Eledone cirrhosa*), musky octopus (*Eledone moschata*), pale octopus (*Octopus pallidus*), variable octopus (*Octopus variabilis*), common octopus (*Octopus vulgaris*), elegant cuttlefish (*Sepia elegans*), and common cuttlefish (*Sepia officinalis*)

The pairwise analysis of dicyemid communities across various marine regions using PERMANOVA and ANOSIM revealed varying degrees of differentiation. Among these, the comparison between the China Sea and Bass Strait showed the highest PERMANOVA *R*^2^ and ANOSIM *R* values of 0.7186 and 0.9497, respectively, indicating a strong and statistically significant difference in their dicyemid communities. On the other hand, both the Atlantic and Mediterranean regions exhibited the lowest PERMANOVA *R*^2^ and ANOSIM *R* values of 0.0368 and 0.107, respectively, indicating minimal variations in their dicyemid communities and suggesting a greater degree of similarity or less ecological differentiation (Fig. 4A). The β-diversity results based on PERMANOVA and ANOSIM were consistent with each other. Regarding the comparison of communities in various host species, PERMANOVA and ANOSIM tests revealed both marked differences and similarities. The maximum PERMANOVA R^2^ and ANOSIM R values were 0.9191 and 1.00, respectively, between *C*. *macropus* and *S*. *elegans*. These values indicate a very robust and statistically significant distinction in their dicyemid communities, suggesting a robust differentiation in the dicyemid populations associated with these hosts. However, the lowest values for both metrics (PERMANOVA *R*^2^ = 0.0254 and ANOSIM *R* = 0.0009) were between *E*. *moschata* and *C*. *macropus*, suggesting negligible variations in their dicyemid communities. This finding indicates a high degree of similarity in the dicyemid profiles of these hosts (Fig. 4B). Moreover, the results from the pairwise comparisons of ANOSIM and PERMANOVA based on the Bray–Curtis dissimilarity revealed notable findings. The greatest *R*^2^ correlation from PERMANOVA, at 0.7106, occurred between the China Sea and Bass Strait regions. In contrast, the lowest *R*^2^, at 0.034, was between the Atlantic and Mediterranean regions. For ANOSIM, the maximum *R* correlation was 0.3786 between the Mediterranean and Bass Strait, while the lowest *R* was 0.033 between the Atlantic and China Sea. Additionally, the analysis of dicyemid diversity based on host species reported the highest *R* value of 0.7784 between *O*. *pallidus* and *A*. *marginatus*; the lowest was − 0.0262 between *C*. *macropus* and *O*. *vulgaris* (Supplementary Fig. S7).

#### Local scale

The α-diversity on a local scale, considering the Atlantic and Mediterranean regions and based on a wide range of indices (ACE, Shannon, Simpson, and InvSimpson), found no marked differences in the dicyemid community diversities across various hosts (Supplementary Fig. S8). However, remarkable geographical differences in dicyemid communities were observed between the Canary Islands, the Northeast Atlantic, and the Western Mediterranean, with the latter showing the highest values across a majority of the tests conducted (Supplementary Fig. S9). Only the evenness metrics did not differ significantly between these regions (Supplementary Fig. S9; *P* < 0.05). The results of the β-diversity analysis, using weighted UniFrac distances, indicated a marked correlation in the dicyemid populations based on cephalopod hosts and marine environments (Supplementary Fig. S10; *P* < 0.05).

## Discussion

Dicyemids were discovered by the Italian Filippo Calvolini in 1787; since then, their enigmatic lifestyle and unresolved classification remain one of the major issues (Aruga et al. 2007; Awata et al. 2006; Catalano 2012; Drábková et al. 2022; Erdl 1843; Furuya and Souidenne 2019; Kölliker 1849; Krohn 1839; Lu et al. 2019; Nouvel 1947; van Beneden 1876, 1882). In this study, we present *18S rDNA* screening of dicyemid biodiversity among coleoid cephalopods employing an amplicon sequencing approach for the first time. We investigated the prevalence and diversity of dicyemids across four geographic regions (Northeast Atlantic, Mediterranean, China Sea, and Bass Strait) and in 24 cephalopod species. For over a decade, the operational taxonomic units (OTUs) have served as the output data from rDNA amplicon sequencing (Kopylova et al. 2016; Schmidt et al. 2014; Westcott and Schloss 2015). Later, a more accurate ASV-based method was introduced (Amir et al. 2017; Callahan et al. 2016, 2017; Edgar 2016; Eren et al. 2013, 2015; Tikhonov et al. 2015), providing enhanced sensitivity and specificity than OTUs, reflecting true biological variability, and distinguishing among the existing ecological patterns. Using the ASV approach, we observed variations in the prevalence rate of dicyemid infections in each cephalopod host, along with differences in dicyemid prevalence, ranging from 0 to 100% among various marine environments. This finding aligns with previous research that reported broad variability in the prevalence rates of dicyemid species based on morphology and *COI* sequencing. For instance, Catalano et al. (2014) reported that the prevalence of dicyemid infections in various cephalopods ranged from 24% to 100%, and was influenced by factors, such as host size, life history, geographical locality, and competition among parasitic species. Similarly, our results indicated that the prevalence rate of dicyemid infections varied significantly between samples from the examined areas and host species. However, we extended our genetic analysis with the results of the dicyemid community diversity structuring, also revealing differences between specific hosts and areas.

### High prevalence among certain hosts and new host records in the Pacific

Our results showed that the prevalence rate of dicyemids in the Atlantic and Mediterranean species of *O*. *vulgaris*, *S*. *officinalis*, and *E*. *moschata*; in the North Pacific, *O*. *variabilis* was considerably greater compared with the other hosts (Table 1). Although little data are available on the Pacific *O*. *variabili*s, results on the Atlantic and Mediterranean species agreed with previous studies. Gestal et al. (1997) reported a 100% prevalence of dicyemid parasites in *O*. *vulgaris* from Northwestern Spain. Drábková et al. (2019) found a high prevalence of dicyemid parasites in *S*. *officinalis*, with remarkable genetic diversity across different Mediterranean subpopulations. A relatively high number of infections have also been documented in *E*. *moschata* and *E*. *cirrhosa*, particularly with the dicyemid species *Dicyema moschatum* and *Dicyemennea eledones* (Drábková et al. 2021; Souidenne et al. 2016). These two octopuses are among the most frequent benthic cephalopods in the Mediterranean Sea and the North East Atlantic Ocean. Both species share similar ecological habitats, which likely facilitates the exchange and enhanced prevalence of dicyemid parasites within these hosts.

*Octopus pallidus* inhabiting similar temperate waters in the southern hemisphere also revealed a high dicyemid prevalence. On the contrary, low dicyemid prevalence occurred in some hosts from the North Pacific Ocean, like *Abdopus aculeatus* and *Octopus laqueus*. Surprisingly, *Sepioteuthis lessoniana* was also rarely infected, even though it is known to infect dicyemids; only 1/11 individuals were infected (Furuya and Tsuneki 2005). Several host species were even negative for the presence of dicyemids (*Euprymna scolopes*, *Metasepia tulbergi*, and *Uroteuthis duvauceli*), which may suggest that dicyemid prevalence may be lower in tropical areas (cfFinn et al. 2005; Hochberg 1990). However, the high temperature and humidity conditions during sampling and the short-term storage of these samples in ethanol prior to their transport to our laboratory cannot be ruled out as a cause for sample degradation. In some cases, the number of examined samples per host species was very limited.

To date, ≥ 80 cephalopod species have been identified as hosts for > 140 dicyemid species across various major oceanic regions such as the Pacific Ocean (encompassing the Okhotsk Sea, Sea of Japan, Western and Eastern North Pacific, and South Pacific), the Indian Ocean (including the North Indian Ocean), the Atlantic Ocean (covering the Western and Eastern North Atlantic, the Mediterranean Sea, and the Gulf of Mexico), and the Antarctic Ocean (Castellanos-Martinez et al. 2016; Catalano 2013a, b; Furuya 1999, 2016, 2018; Furuya and Moritaki 2022; Furuya and Tsuneki 2005; McConnaughey 1951; Roumbedakis et al. 2018). The present study identified eight new cephalopod host species from the Pacific Ocean. They included *Amphioctopus aegina*,* A*. *marginatus*,* A*. *ovolum*, *Cistopus taiwanicus*, *Octopus incella*, *O*. *laqueus*, *Uroteuthis chinensis*, and *Nototodarus hawaiensis*. These cephalopods are predominantly benthic, with the exception of *U*. *chinensis* and *N*. *hawaiiensis*, which are types of semi-pelagic squids (Roper et al. 1984). The occurrence of dicyemids in squids is particularly intriguing as they are rarely associated with dicyemids due to their pelagic lifestyle. This finding indicates that dicyemids may be found in ecologically diverse host groups and that their host spectrum is wider than expected.

### Dicyemid diversity structured by geographic distribution and host range

Comparison of genetic data and morphology showed that, at least in some cases, the delimitation of dicyemid species or genera based only on morphological traits may be insufficient (Eshragh and Leander 2014; Nakajima et al. 2024). Similar to Nakajima et al. (2024), our results failed to recover the monophyletic genera *Dicyema* and *Dicyemennea*, which have been traditionally recognized morphologically. Additionally, Eshragh and Leander (2014) utilized molecular phylogenetic tools to analyze the *18S rDNA* sequences from 34 dicyemid samples representing eight morphospecies, demonstrating that they represent a single or closely related species exhibiting considerable morphological variability within each cephalopod host. In our species delimitation analyses, including a wider range of dicyemid *18S rDNA* sequences, these samples also formed a single genetic lineage. This finding raises an intriguing hypothesis that the morphological variations observed in the calotte shapes between *Dicyema* and *Dicyemennea* (Eshragh and Leander 2014) may result from phenotypic plasticity rather than true genetic differentiation. Aruga et al. (2007) provided evidence that the expression of the development-associated genes *Pax6* and *Zic*, along with accelerated molecular evolution and highly reduced intron sizes in *Dicyema*, may contribute to its morphological variability. These findings support the possibility that developmental plasticity, rather than permanent genetic differences, plays a role in shaping *Dicyema* morphology, potentially in response to environmental factors. The *18S rDNA* gene typically reflects deeper divergence among species and is less variable than, for example, the *COI* genetic marker usually used for species distinction. However, in general, we discovered a surprisingly broad diversity among dicyemid types based on *18S rDNA* amplicon sequencing. Further studies incorporating genomic and transcriptomic data are needed to properly distinguish phenotypic plasticity from genetic differentiation in dicyemids.

Even though our sample size was a fraction of the global cephalopod biodiversity (4%, 3%, and 1.7% of the total octopod, sepiid, and teuthid diversity, respectively), the number of identified dicyemid types was relatively high. Thus, according to our study, the number of known dicyemid species markedly underrepresents their true diversity. Studies that explored the host specificity of dicyemid parasites found that while certain cephalopod hosts are exclusively infected by a single dicyemid species, others can harbor multiple species (Catalano et al. 2014; Furuya and Souidenne 2019; Nakajima et al. 2024). For instance, *O*. *berrima* and *O*. *kaurna* were each infected by a single dicyemid species, indicating a high degree of host specificity. In contrast, species like *S*. *apama* and *S*. *novaehollandiae* hosted multiple dicyemid species (Catalano et al. 2014). Similarly, our analysis of the dicyemid type distribution revealed that certain hosts predominantly showed single dicyemid types across a majority of the individuals. For example, in the Bass Strait, although *O*. *pallidus* harbored six types in total, it demonstrated a single dicyemid type across a majority of the individuals. In the China Sea, *O*. *variabilis* and *A*. *marginatus* exhibited single dicyemid types. On the contrary, several hosts showed multiple dicyemid types: *O*. *vulgaris* and *S*. *officinalis* in the Atlantic; and *E*. *cirrhosa*, *E*. *moschata*, *S. elegans*, and *S*. *officinalis* in the Mediterranean. Specifically, we found 46 dicyemid types in *S*. *officinalis*, which contrast with only four species that have been recorded previously: *Dicyemennea gracile*, *Pseudicyema truncatum*, *Dicyema whitmani*, and *Microcyema vespa* (Furuya and Souidenne 2019). Of these, *P*. *truncatum* was the most commonly observed species with the greatest prevalence (Furuya and Souidenne 2019).

Our results confirmed that > 1 dicyemid genetic type can occur in almost all of the host species screened (Fig. 2; Table 1). Sometimes, several dicyemid types occurred within a single cephalopod individual (Supplementary Figs. S5, S8), a finding in agreement with previous studies (Furuya 1999; Furuya et al. 2003). On the contrary, the absence of dicyemids in some species analyzed in this study (e.g., from the China Sea or Hawaii) is questionable, likely due to insufficient sampling depth in most cases (only one-to-six individuals examined). However, better sampled species from the same area revealed the presence of dicyemids, even if usually at a low prevalence (Table 1). These results imply that the host specificity of dicyemid parasites varies markedly among various cephalopod hosts, possibly influenced by ecological factors and the competitive interactions among dicyemid species within the same host.

The results of α-diversity, based on a broad range of diversity indices (Fig. 3), revealed that dicyemid α-diversity in the Bass Strait (Australia) is markedly higher than that in the Atlantic, Mediterranean, and China Seas. This finding is crucial, considering that our study examined only a single host species (*O*. *pallidus*) in this region. High values for Faith’s PD suggest that the dicyemid community in the Bass Strait includes a broad range of evolutionary lineages (Fig. 2), indicating a deep and diverse evolutionary history (Faith 1992; Frishkoff et al. 2014). Moreover, the ACE index indicates a high, estimated species richness in the Bass Strait, accounting for several rare species. Environmental factors in the Bass Strait could foster distinct genetic lineages within the dicyemid community, for example, by mixing faunas between the east (Indian Ocean) and west (Pacific Ocean). Additionally, a few other studies have highlighted the broad diversity of dicyemid species in Australia. For example, Catalano et al. (2013) described five new species isolated from various cephalopod hosts, such as cuttlefish and octopus, indicating a rich and diverse dicyemid fauna in this region. However, in our study, as only a single host species was analyzed in the Bass Strait, the high α-diversity observed could reflect the prevalence of parasite diversity within that host. It may not truly represent the dicyemid diversity in this region and could inflate diversity estimates compared to regions with multiple host species. Therefore, further studies monitoring more cephalopod hosts in this region, as well as other underexplored areas, are still needed to draw firm conclusions. Nevertheless, since a majority of the dicyemid research has focused on the northern hemisphere, the information gained here on *O*. *pallidus* contributes to the sparse work on dicyemid infections in the southern hemisphere (Catalano 2013a, b; Catalano and Furuya 2013; Finn et al. 2005).

Linking genetic types to morphological species is challenging in the case of dicyemids due to the scarcity of published data, as can be seen from our analyses (Fig. 2). The multiple lineages seen in the phylogenetic tree constructed based on the *18S rDNA* sequences do not contain any reference suggesting great hidden dicyemid diversity. The unfortunate absence of a morphological description of the sequenced species in our study is due to the fact that the samples were generally either collected under field conditions without fixation of dicyemid individuals for slide mounting or shipped to us as frozen host tissues. Additionally, the limited sample sizes for certain hosts, such as *Sepia elegans*, may further constrain the reliability of species linkage identified within these lineages. In comparison with the current literature that describes the co-occurrence of dicyemid species in cephalopod hosts with the dicyemid types identified (Supplementary Table S6), we can very tentatively match the results presented herein to the respective types in certain cephalopod hosts. For instance, the widespread 058_Porto03 type, similar to the previously described *Dicyemennea lameerei*, occurs in *E*. *cirrhosa* and *O*. *vulgaris* but also in other hosts. The 069_Tyrh01 type found in *C*. *macropus*,* O*. *vulgaris*, and *E*. *moschata* may correspond to *Dicyema paradoxum* (Catalano 2012; Nouvel 1947). The 013_Catalonia01 type, commonly found exclusively in *Sepia elegans*, suggests a match with either *D*. *schulzianum* or *D*. *macrocephalum* (Nouvel 1947). Contrary to these cases, the 089_Vietnam07 type found in *Sepioteuthis lessoniana* did not correspond to *Dicyema orientale* previously reported in this cephalopod, suggesting that it could be *Dicyema koshidai*, the only other known dicyemid from this host species (Furuya et al. 2004). The lack of resolution in this area clearly signifies the need for a coordinated effort to revise the dicyemid taxonomy with the inclusion of molecular data.

Certain cephalopod species demonstrate higher prevalence and dicyemid diversity, whereas others do not show any infections (Castellanos-Martínez et al. 2011; Catalano et al. 2014; Finn et al. 2005). There are also notable variations in the number of dicyemid types among individuals within the same species, further emphasizing the complex interplay between host specificity and geographic location in shaping dicyemid diversity (Catalano et al. 2014; Finn et al. 2005; Furuya 2018; Furuya and Souidenne 2019; Nakajima et al. 2024). Similarly, β-diversity results using PERMANOVA revealed a significant geographic component that explains ≤ 52% of the diversity variation (*R*^2^ = 0.52). Additionally, when considering the cephalopod host as a factor, PERMANOVA explained 72% (*R*^2^ = 0.72) of the variations in diversity, and ANOSIM indicated a significant separation with an *R* value of 0.70. The importance of geographic distance was also clearly demonstrated by the pairwise analysis of dicyemid communities, with the strongest variation occurring between the China Sea and the Bass Strait (PERMANOVA *R*^2^ of 0.7186 and ANOSIM R of 0.9497), compared to the Atlantic and Mediterranean regions (PERMANOVA *R*^2^ of 0.0368 and ANOSIM *R* of 0.107). These results suggest that geographic location markedly influences dicyemid diversity, with certain regions exhibiting more distinct community structures than others.

In addition to the influence of geography on dicyemid communities, some species are observed in a single host, with most types exhibiting a narrow host specificity, involving only a few hosts (Catalano and Furuya 2013; Nakajima et al. 2024). For example, Catalano and Furuya (2013) found that *Dicyema calamaroceum* was only present in *Sepioteuthis australis*, whereas *Dicyema pyjamaceum* was exclusively found in *Sepioloidea lineolata*, despite the hosts occurring within the same geographical regions. Similarly, the β-diversity results of our study indicated significant differences between the hosts. The greatest variations were observed between* C*. *macropus* and *S*. *elegans* (PERMANOVA *R*^2^ = 0.9191 and ANOSIM *R* = 1.00) despite their co-occurrence in the Mediterranean. In contrast, several dicyemid types demonstrated a geographically wide distribution and a broad host spectrum (033_Dic01, 058_Porto03, 069_Tyrh01, and 083_Viet01), indicating previously undescribed dicyemid universality. These findings imply the importance of host specificity in shaping dicyemid communities, suggesting that host identity plays a more crucial role than geographic location in determining the composition of these communities.

To conclude, this study represents the first use of amplicon sequencing to explore dicyemid biodiversity. Our findings indicated a significant variability in dicyemid prevalence and diversity influenced by host species and geographic location. Greater prevalence rates in specific cephalopods, coupled with the discovery of new host species in the Pacific, highlight the adaptability and extensive range of dicyemids. The use of amplicon sequencing has uncovered substantial genetic diversity that challenges traditional morphology-based classifications, suggesting that the true diversity of the dicyemid species is considerably underrepresented. The high α-diversity observed in the Bass Strait, despite being dominated by a single dicyemid type, illustrates the importance of genetic variability and evolutionary history in shaping dicyemid communities. Our analyses indicate that geographic factors and host affiliations play critical roles in structuring these communities, with significant implications for understanding host–parasite dynamics. Despite our limited sampling, the obtained insights advance our knowledge of dicyemid biodiversity and emphasize the need for continued genetic research to unravel the complexities of dicyemid classification and ecology.

## Supplementary Information

Below is the link to the electronic supplementary material.

Supplementary file1 (DOCX 2245 KB)

Supplementary file2 (XLSX 195 KB)

Supplementary file3 (FASTA 255 KB)

## Data Availability

The complete set of genetic data produced in this study, including raw Illumina reads, is deposited in GenBank under the BioProject number PRJNA1274912.
