# Supplementary material for: Amplicon sequencing reveals the cryptic diversity in the dicyemid parasites of coleoid cephalopods sampled from the Atlantic and Pacific Oceans
Source: Mar Life Sci Technol. 2026 Feb 17;8(1):16–31. doi: 10.1007/s42995-026-00353-w (PMC12953835; doi:10.1007/s42995-026-00353-w)
Supplement: Supplementary file 1 — Supplementary file1 (DOCX 2245 KB) [file 42995_2026_353_MOESM1_ESM.docx]

**Supplementary material for the article:**

**Amplicon sequencing reveals the cryptic diversity in the dicyemid parasites of coleoid cephalopods sampled from the Atlantic and Pacific oceans**

Tijana Cvetković^1,2*^, Masoud Nazarizadeh^1,3,*^, Tereza Koudelková^1,3,*^, Fedor Lishchenko^4,5^, Yen H.T. Dinh^5^, Eduardo Almansa^6^, Hannah Osland^7^, Tomáš Scholz^1,3^, Zdeněk Lajbner^8^, Qiaz Q.H. Hua^9^, Marie Drábková^10^ & Jan Štefka^1,3,#^

^1^ Institute of Parasitology, Biology Centre CAS, České Budějovice 370 05, Czech Republic

^2^ Department of Biological Sciences, University at Buffalo, Buffalo, NY 14260, USA

^3^ Faculty of Science, University of South Bohemia, České Budějovice 370 05, Czech Republic

^4^ A.N. Severtsov Institute of Ecology and Evolution of the RAS, Moscow 119071, Russia

^5^ Coastal Branch of the Joint Vietnam - Russia Tropical Science and Technology Research Center, Nha Trang 650000, Vietnam

^6^ Centro Oceanográfico de Canarias, Instituto Español de Oceanografía (IEO), CSIC, Santa Cruz de Tenerife 38180, Spain

^7^ College of Science and Technology, Temple University, Philadelphia, PA 19122, USA

^8^ Physics and Biology Unit, Okinawa Institute of Science and Technology Graduate University, Okinawa 904-0495, Japan

^9^ Environment Institute, Department of Ecology and Evolution, The University of Adelaide, SA 5005, Australia

^10^ Faculty of Science, University of Hradec Králové, Hradec Králové 500 03, Czech Republic

* Equally contributed

^#^ For correspondence. E-mail: jan.stefka@prf.jcu.cz

**Supplementary material:**

**A. Material used in this study and library preparation:**

**Supplementary Figure S1:**


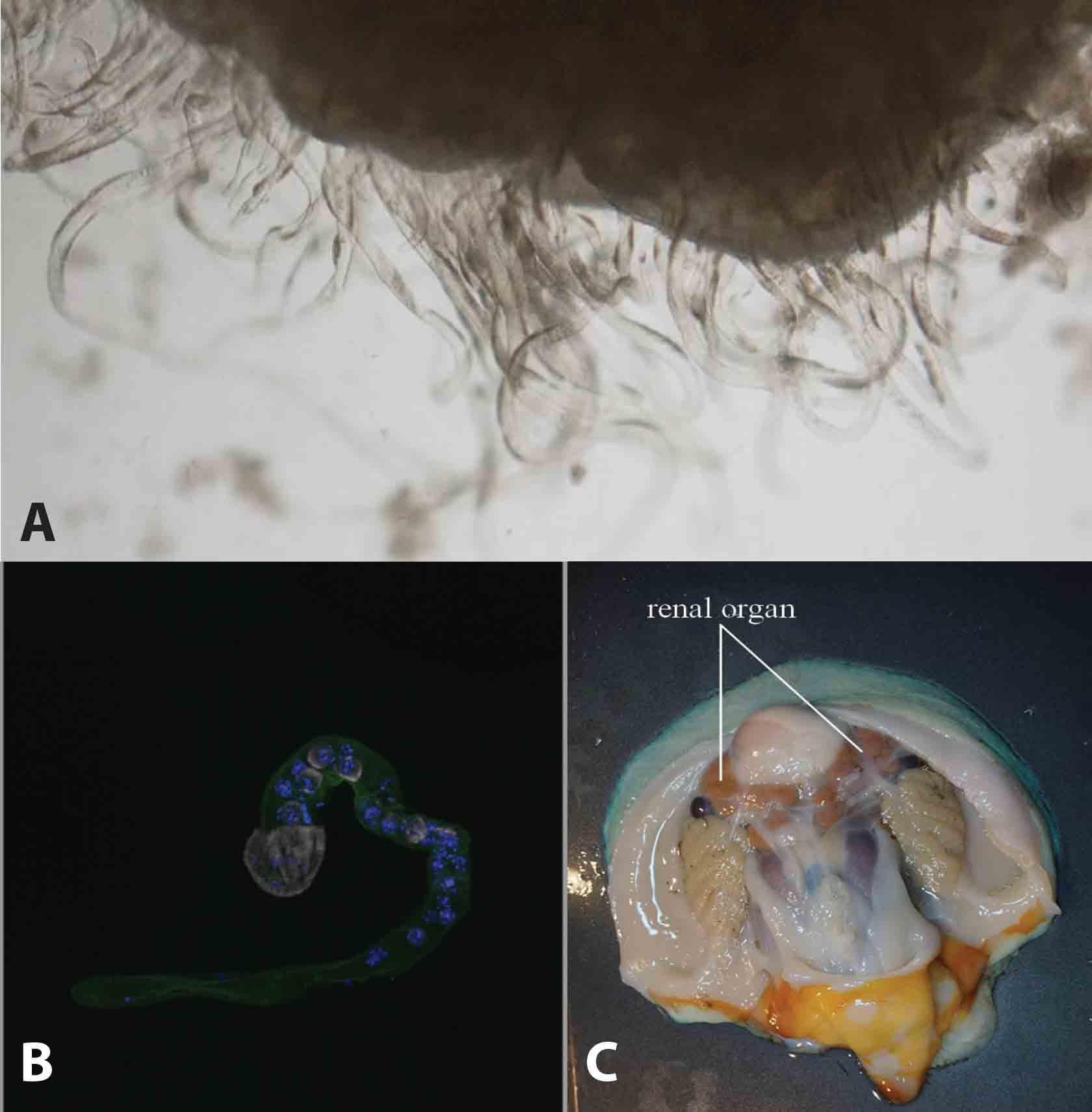


**Fig. S1:** **A.** Dicyemid individuals attached to a renal organ of cephalopod; **B.** Confocal microscopy of *Dicyema moschatum*: DAPI + phalloidin (actin; green) + beta-tubulin (white); **C.** Dissected octopus *Eledone moschata*. Photos by Marie Drábková.

**Verifying the host species:**

Gene marker cytochrome c oxidase I (*COI*) was used to determine the host species by PCR (see the following Tab. A.1). The reaction contained: 1 µL of DNA sample, 1 µL of forward primer (5pM, F1490, Folmer et al., 1994; sequence in Tab. A.1), 1 µL of reverse primer (5pM, H7005, Hafner et al., 1994; sequence in Tab. 1 or 5pM, H2198, Folmer et al., 1994; sequence in Tab. A.1), 2 µL of buffer (PCR blue buffer, Top-bio), 0,5 µL of nucleotides (dNTPs 10mM, ThermoFisher), 0,2 µL of Taq polymerase (Top-bio) and 14,3 µL of H_2_O.

| **Loci** | **Name** | **Sequence of the primer 5'-3'** | **Forward / Reverse** |
| --- | --- | --- | --- |
| host’s COI | F1490 | GGTCAACAAATCATAAAGATATTGG | F |
|  | H7005 | CCGGATCCACANCRTARTANGTRTCRTG | R |
|  | H2198 | TAAACTTCAGGGTGACCAAAAAATCA | R |

**Table A.1:** Primer sequences for the COI PCR amplification.

For the specific thermocycler setup see Tab. A.2. PCR products were visualized by gel electrophoresis (1% agarose, 1KB GeneRuler ladder ThermoFisher), enzymatically cleaned (0,5 µL exo I nuclease Top-bio, 2 µL FastAP, 2,5 µL H2O), and Sanger sequenced using PCR primers in a commercial laboratory (Seqme, CZ).

| **Loci** | **Initial denaturation** | **No. of cycles** | **Denaturation** | **Annealing** | **Elongation** | **Final elongation** |
| --- | --- | --- | --- | --- | --- | --- |
| Host’s COI | 94° C  5 minutes | 30 | 92° C  1 minute | 52° C  1 minute | 72° C  1 minute | 72° C  5 minutes |

**Tabble A.2:** Thermocycler configuration for the COI PCR amplification.

Species of host was determined by comparison to NCBI GenBank database.

**Amplicon DNA library preparation:**

Amplicon library was prepared following a slightly modified Illumina protocol (Illumina 16S metagenomic sequencing library preparation, 2017). A two-stage PCR protocol was economically efficient, allowing potential sharing of barcoded oligos between different projects (opposite to one-stage PCR).

**Designing oligonucleotides:**

Final amplicon library has low complexity and consists of millions of the same or highly similar reads. This could be a potential problem in the cluster identification and color matrix estimation for the sequencing machine, as the same signal flashes causing sequencing failure (de Muinck et al., 2017). One of the most common solutions is adding a spike-in of PhiX DNA to diversify the library and therefore prevent the signal from clashing. The downside of this approach is the fact that adding a substance that is not of our interest takes up space on the sequencing lane, lowering the amount of obtained data.

To avoid losing data to PhiX spike-in, we decided to use heterogeneity primers, i.e. inserting short spacers (0-4 nucleotides long) to our construct, similarly to Fadrosh et al., 2014. These spacers are random nucleotides inserted in front of the primer itself but also after the reading site (reading primers Rd1 SP, Rd2 SP). The construct added in the first round PCR (referred to as Amplicon PCR) consists of the primer itself (primers 926R and 515F-Y by Parada et al., 2016), spacer, and the overhang, necessary for binding the indices and adapters in the second-round PCR (Fig. A.1). In the second round PCR (referred to as Index PCR) the construct consisting of the Illumina adapter site, also known as P5 and P7, is added together with the index and an overhang that binds to the overhang from the Amplicon PCR (Fig. A.1). We used a dual indexing approach for multiplexing up to 384 samples for one sequencing run, which significantly reduced the cost of sequencing. To determine the best molarity of used primers, Bioanalyzer was run to check for primer-dimers, the best molarity of used primers, and other potentially unwanted activities.

**
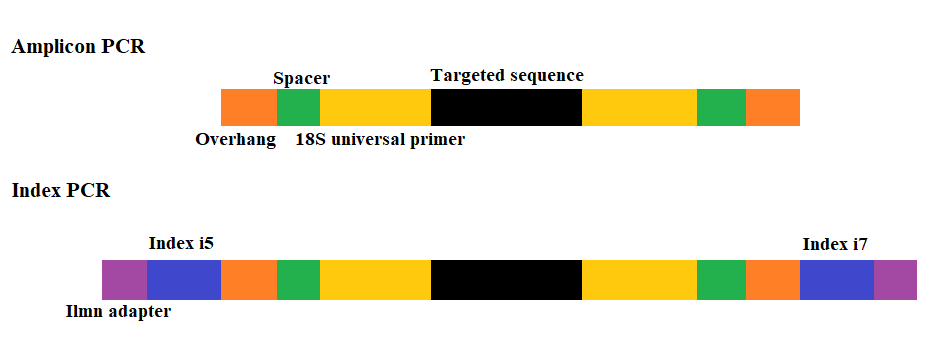
**

**Fig. A.1:** Visual representation of the construct added to the Targeted sequence.

**Amplicon PCR:**

Amplicons were prepared in two batches. For the first library, amplicon PCR was carried out for 185 samples in duplicates to control for amplification bias, plus negative controls, i.e. 374 reactions were done in total for the first prepared library representing 126 unique biological samples. The second library was prepared using 252 samples representing 101 unique samples. The required DNA template concentration for every sample was min. 4 ng/µL, but the average was around 12 ng/µL. Every reaction consisted of 12,5 µL KAPA HotStart ReadyMix (Roche), 5 µL 1µM reverse primer (for the sequences see Tab. A.3), 5 µL 1µM forward primer (for the sequences see Tab. A.3), and 2,5 µL of the sample. For a description of the thermocycler setup see Tab. A.4.

| **Loci** | **Name** | **Sequence of the primer 5'-3'** | **Forward / Reverse** |
| --- | --- | --- | --- |
| 16S bacterial/ 18S dicyemid | Ilmn-16S-FW | TCGTCGGCAGCGTCAGATGTGTATAAGAGACAGN(0-4x)GTGCCAGCMGCCGCGGTAA | F |
|  | Ilmn-16S-RV | GTCTCGTGGGCTCGGAGATGTGTATAAGAGACAGN(0-4x)CCGYCAATTYMTTTRAGTT | R |

**Table A.3:** Primers used for the Amplicon PCR.

| **Loci** | **Initial denaturation** | **No. of cycles** | **Denaturation** | **Annealing** | **Elongation** | **Final elongation** |
| --- | --- | --- | --- | --- | --- | --- |
| 16S bacterial/ 18S dicyemid | 95° C  3 minutes | 25 | 95° C  30 seconds | 55° C  30 seconds | 72° C  30 seconds | 72° C  5 minutes |

**Table A.4:** Thermocycler configuration for Amplicon PCR.

**Clean-up and quantification:**

The clean-up of the PCR reaction was carried out using AMPure XP beads (Beckman): 20 µL of magnetic beads were added to each reaction, incubated for 5 minutes outside of the magnetic stand, followed by 2-minute incubation on the magnetic stand. These steps were followed by two ethanol washes (80% EtOH) and then incubating washed beads in 52,5 µL of H_2_O for 2 minutes outside the magnetic stand and for 2 minutes on the magnetic stand. Finally, 50 µL of cleaned-up PCR product was transferred to new tubes.

In order to verify the success of PCR and clean-up, all samples were measured on a Qubit fluorometer using a dsDNA High Sensitivity kit. To verify that desired product was obtained, gel electrophoresis was done as well; specifically we used 1,5% agarose gel with GelRed (Biotium), GeneRuler 100 bp Plus DNA (ThermoFisher) ladder, and to load samples 6x Loading Dye (ThermoFisher). Only products with visible bands and/or concentrations above 0,2 ng/µL were used in further steps.

**Index PCR:**

For the index PCR of the first prepared library, most of the duplicates from the first reaction were merged, and only about one-third of the duplicates were kept separate (in order to compare the sequencing results for the duplicates, to reveal whether any sequencing bias occurs or not). This merging resulted in preparing 216 reactions, instead of the original 370 (374 with negative controls). For the index PCR of the second library, we proceeded with 224 samples out of 252 original samples. The reaction was done as follows: 25 µL KAPA HotStart ReadyMix (Roche), 10 µL H_2_O, 5 µL of the Amplicon PCR product, 5 µL of the 5µM forward index primer, and 5 µL of the 5µM reverse index primer (for sequences of the primers see Tab. A.5; for the sequences of the indices see Tab. A.5.1). For detailed thermocycler setup, see Tab. A.6.

| **Name** | **Sequence of the primer 5'-3'** | **Forward / Reverse** |
| --- | --- | --- |
| S5XX | AATGATACGGCGACCACCGAGATCTACAC(indexS5XX)TCGTCGGCAGCGTC | F |
| N7XX | CAAGCAGAAGACGGCATACGAGAT(indexN7XX)GTCTCGTGGGCTCGG | R |

**Table A.5:** Sequences of the primers used for the Index PCR.

| **Index name** | **Sequence 5'-3'** | **Index name** | **Sequence 5'-3'** |
| --- | --- | --- | --- |
| N701 | TCGCCTTA | N723 | GAGCGCTA |
| N702 | CTAGTACG | N724 | CGCTCAGT |
| N703 | TTCTGCCT | N726 | GTCTTAGG |
| N704 | GCTCAGGA | N727 | ACTGATCG |
| N705 | AGGAGTCC | N728 | TAGCTGCA |
| N706 | CATGCCTA | N729 | GACGTCGA |
| N707 | GTAGAGAG | S502 | CTCTCTAT |
| N710 | CAGCCTCG | S503 | TATCCTCT |
| N711 | TGCCTCTT | S505 | GTAAGGAG |
| N712 | TCCTCTAC | S506 | ACTGCATA |
| N714 | TCATGAGC | S507 | AAGGAGTA |
| N715 | CCTGAGAT | S508 | CTAAGCCT |
| N716 | TAGCGAGT | S510 | CGTCTAAT |
| N718 | GTAGCTCC | S511 | TCTCTCCG |
| N719 | TACTACGC | S513 | TCGACTAG |
| N720 | AGGCTCCG | S515 | TTCTAGCT |
| N721 | GCAGCGTA | S516 | CCTAGAGT |
| N722 | CTGCGCAT |  |  |

**Table A.5.1:** Sequences of the indices.

| **Loci** | **Initial denaturation** | **No. of cycles** | **Denaturation** | **Annealing** | **Elongation** | **Final elongation** |
| --- | --- | --- | --- | --- | --- | --- |
| Illumina index addition | 95° C  3 minutes | 8 | 95° C  30 seconds | 55° C  30 seconds | 72° C  30 seconds | 72° C  5 minutes |

**Table A.6:** Thermocycler configuration for the Index PCR.

**Clean-up and quantification:**

Clean-up of the indexing PCR reaction was carried out using AMPure XP beads (Beckman): 56 µL of magnetic beads were added to each reaction, incubated for 5 minutes outside of the magnetic stand, followed by 2-minute incubation on the magnetic stand. These steps were followed by two ethanol washes (80% EtOH) and then incubating washed beads in 27,5 µL of H_2_O for 2 minutes outside the magnetic stand and 2 minutes on the magnetic stand. Finally, 25 µL of cleaned-up PCR products were transferred to new tubes.

As in the previous PCR, to verify the success of PCR and clean-up, all samples were measured on a Qubit fluorometer using a dsDNA High Sensitivity kit. To verify that the expected product was obtained, gel electrophoresis was done as well, in the same way as described above.

**Library quantification, normalization, and pooling:**

To provide optimal and unbiased sequencing results, all samples were set up to the same concentration before pooling. The first library had a 5 nM concentration, required for sequencing (Norwegian Sequencing Center). We included 126 unique samples (188 samples with duplicates), excluding the samples of poor quality. The second library that had a 7 nM concentration included 101 unique samples (165 samples with duplicates) and was sequenced by Novogene. We used the following formula for molarity calculation: (concentration in ng/µL)/ (660 g/mol × average library size) × 10^6^ = concentration in nM. The average library size was determined to be 660 bp long using Bioanalyzer. The prepared library was sequenced on the MiSeq Illumina machine, using 250 bp paired-end sequencing (Norwegian Sequencing Center, Oslo, Norway, and Novogene, Cambridge, UK).

**B. The results of the bioinformatic analyses:**

**
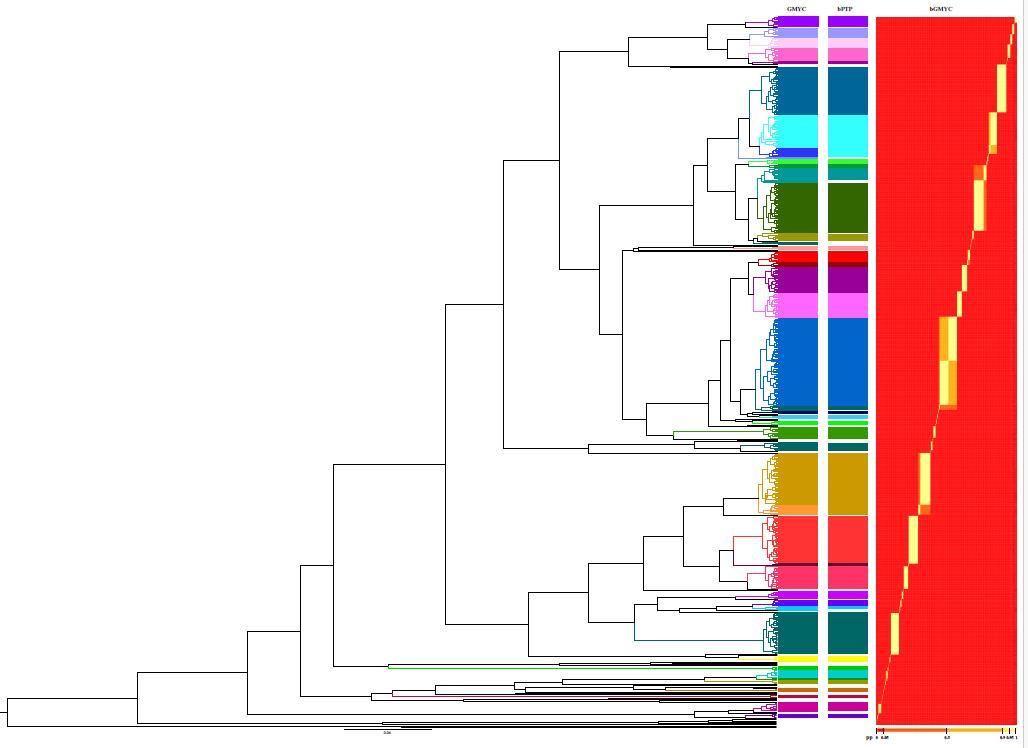
**

**Fig. S2:** Phylogenetic tree of dicyemid types described by species delimitation methods: General Mixed Yule Coalescent (GMYC), and Poisson Tree Process (PTP).


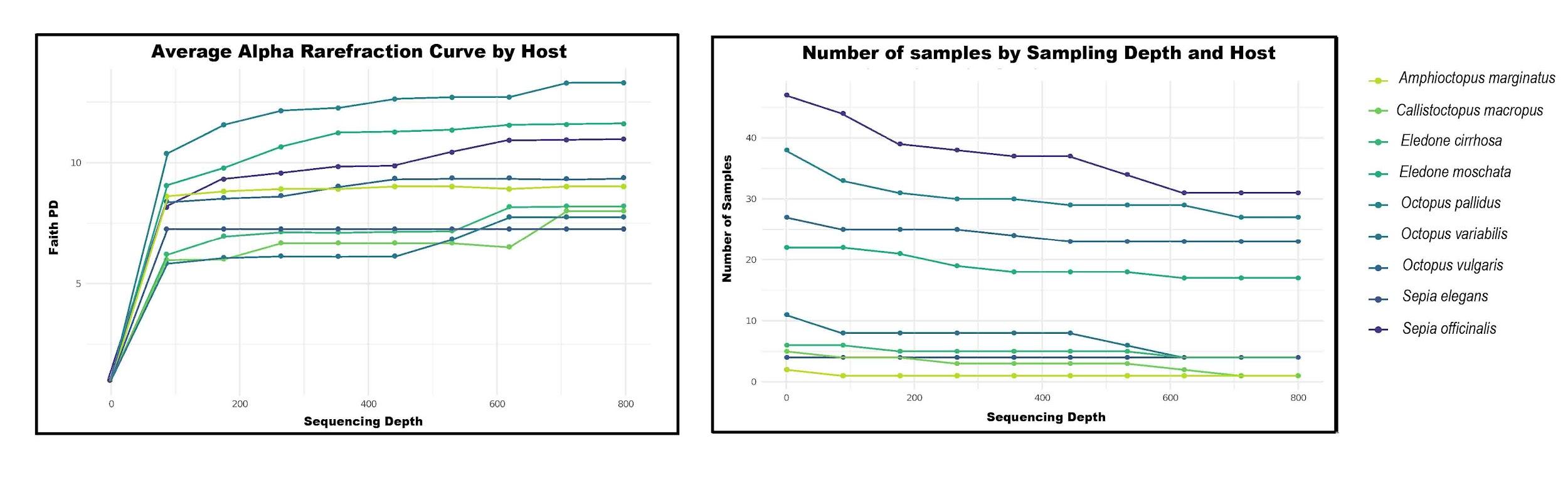


**Fig. S3:** Rarefaction plot and sequencing depth structured by analyzed hosts dispersed globally.

**
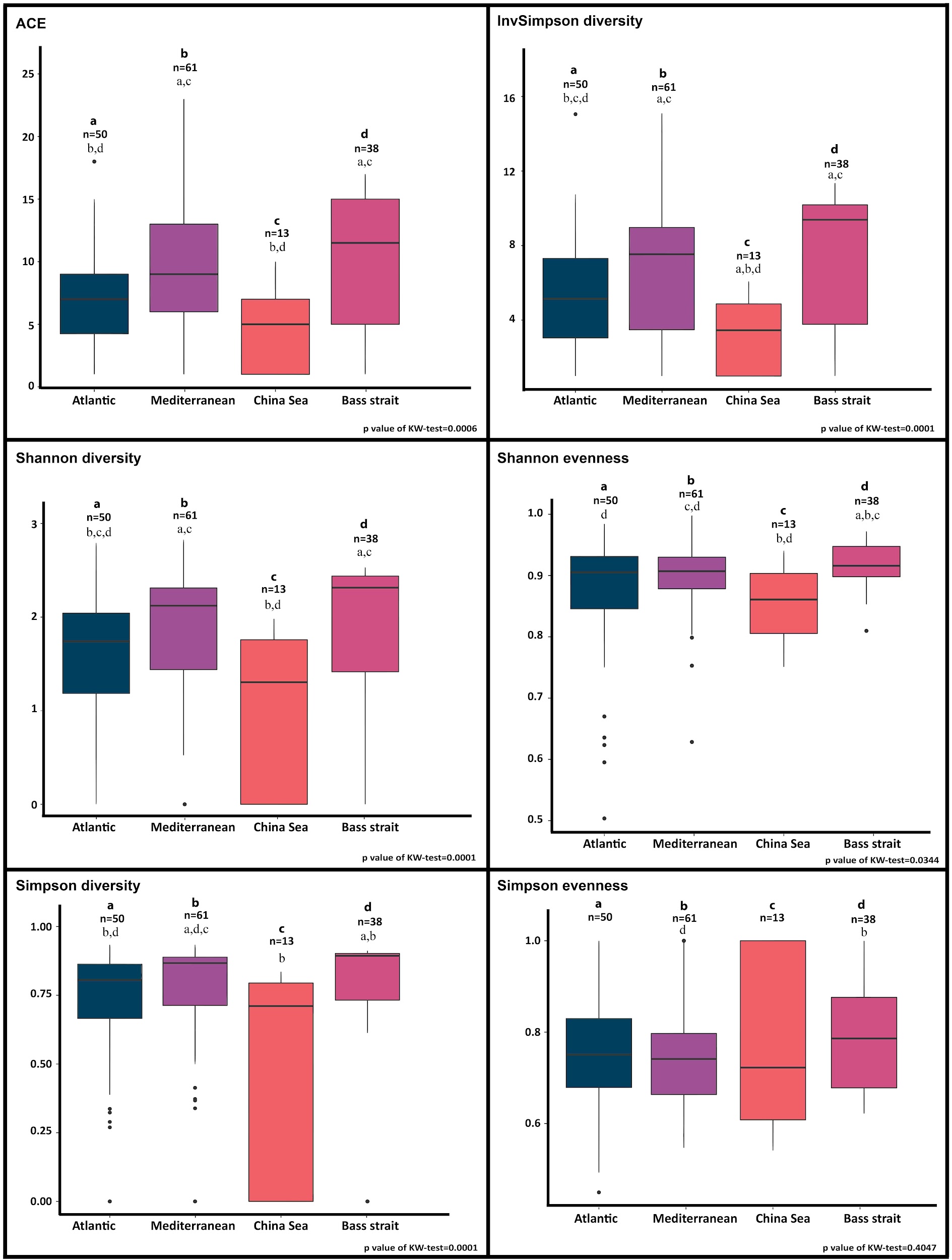
Fig. S4:** Patterns of Alpha (boxplot) diversity of dicyemid communities structured by global geographical regions analyzed in this study (letters below the sample numbers represent localities different from the selected one in a Kruskal–Wallis test).


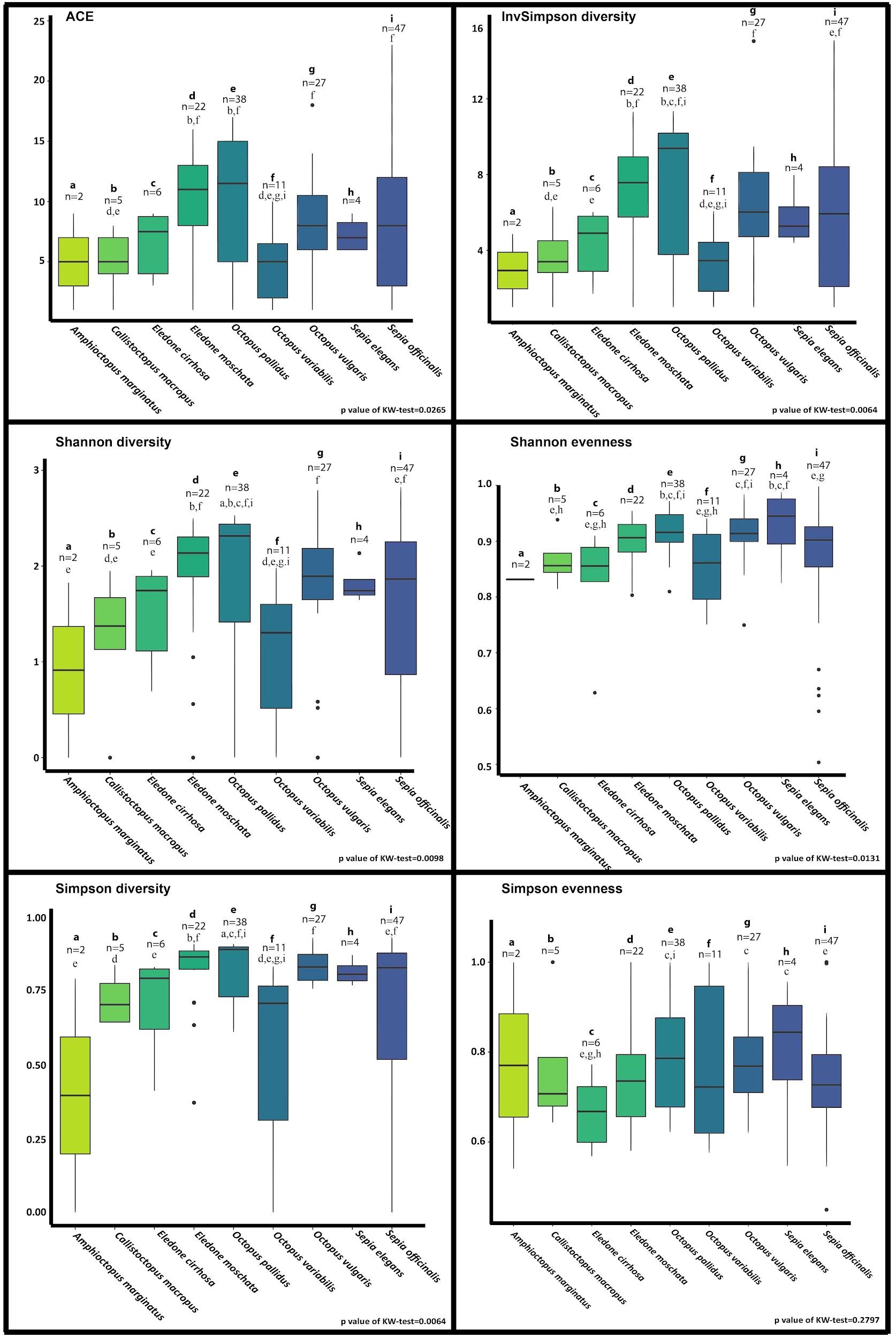


**Fig. S5:** Patterns of Alpha (boxplot) diversity of dicyemid communities structured by analyzed hosts dispersed globally (letters below the sample numbers represent localities different from the selected one in a Kruskal–Wallis test).


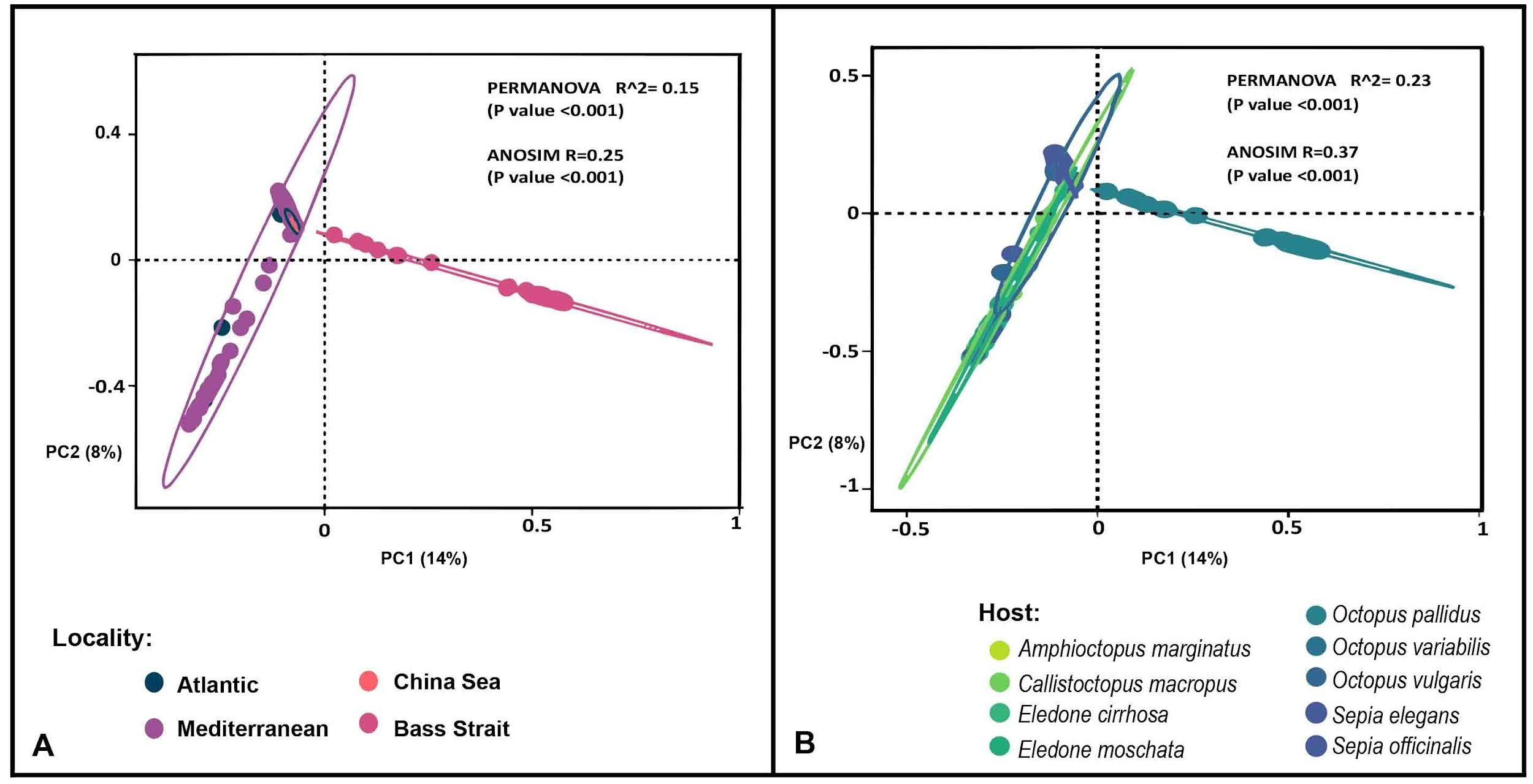


**Fig. S6:** Patterns of beta diversity based on Bray-Curtis dissimilarity in dicyemid communities: **A.** Diversity structured by global geographical regions analyzed in this study; **B.** Diversity structured by host species analyzed across marine environments.

**
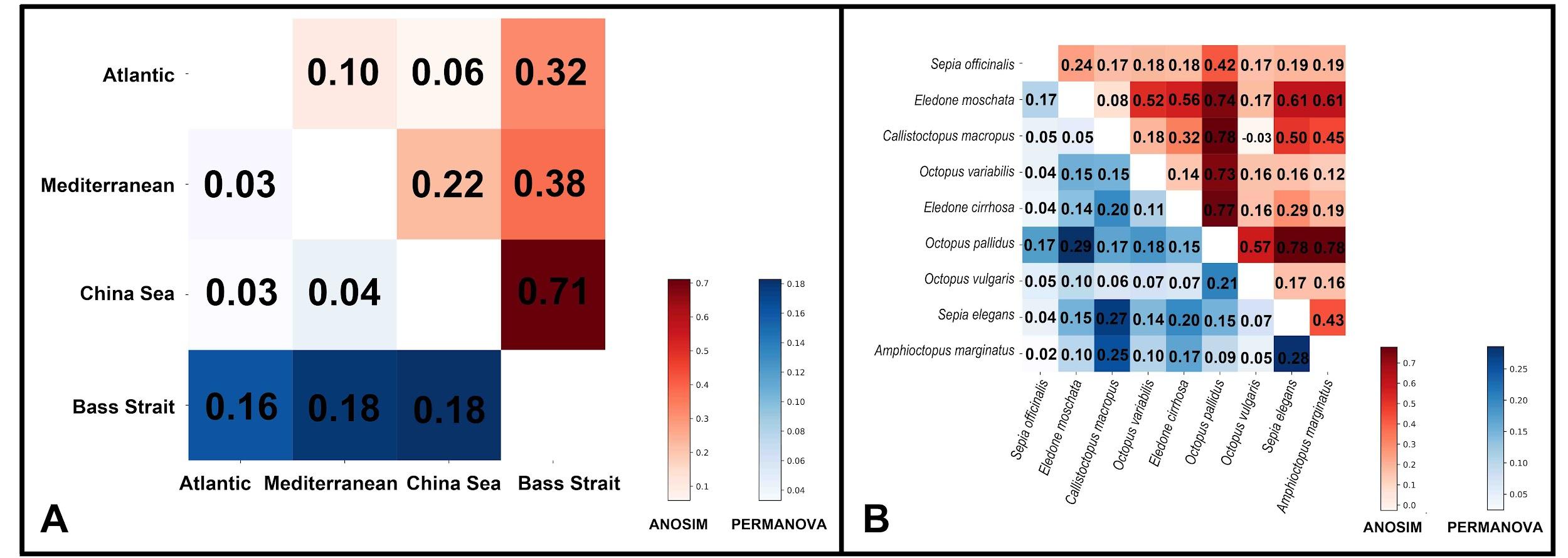
**

**Fig. S7:** Pairwise comparison heatmaps of ANOSIM (upper) and PERMANOVA (lower) from Bray-Curtis Beta Diversity: The numbers indicate the R² correlation from PERMANOVA and the R correlation from ANOSIM. **A.** Structured by global geographical regions analyzed in this study; **B.** Structured by host species analyzed across global locations.

**
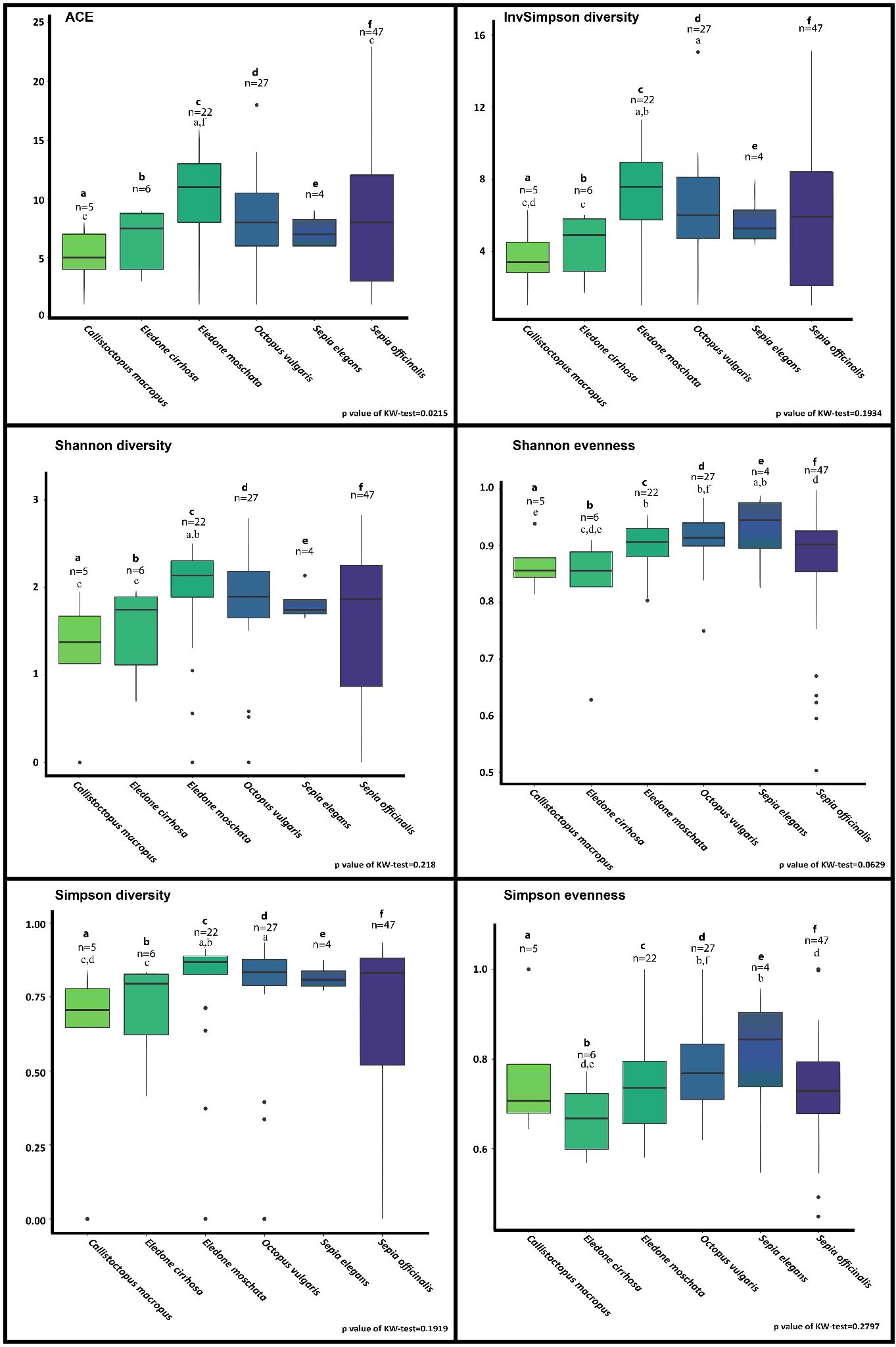
Fig. S8:** Patterns of Alpha (boxplot) diversity of dicyemid communities structured by hosts occurring in the Atlantic and Mediterranean regions analyzed in this study (letters below the sample numbers represent localities different from the selected one in a Kruskal–Wallis test).

**
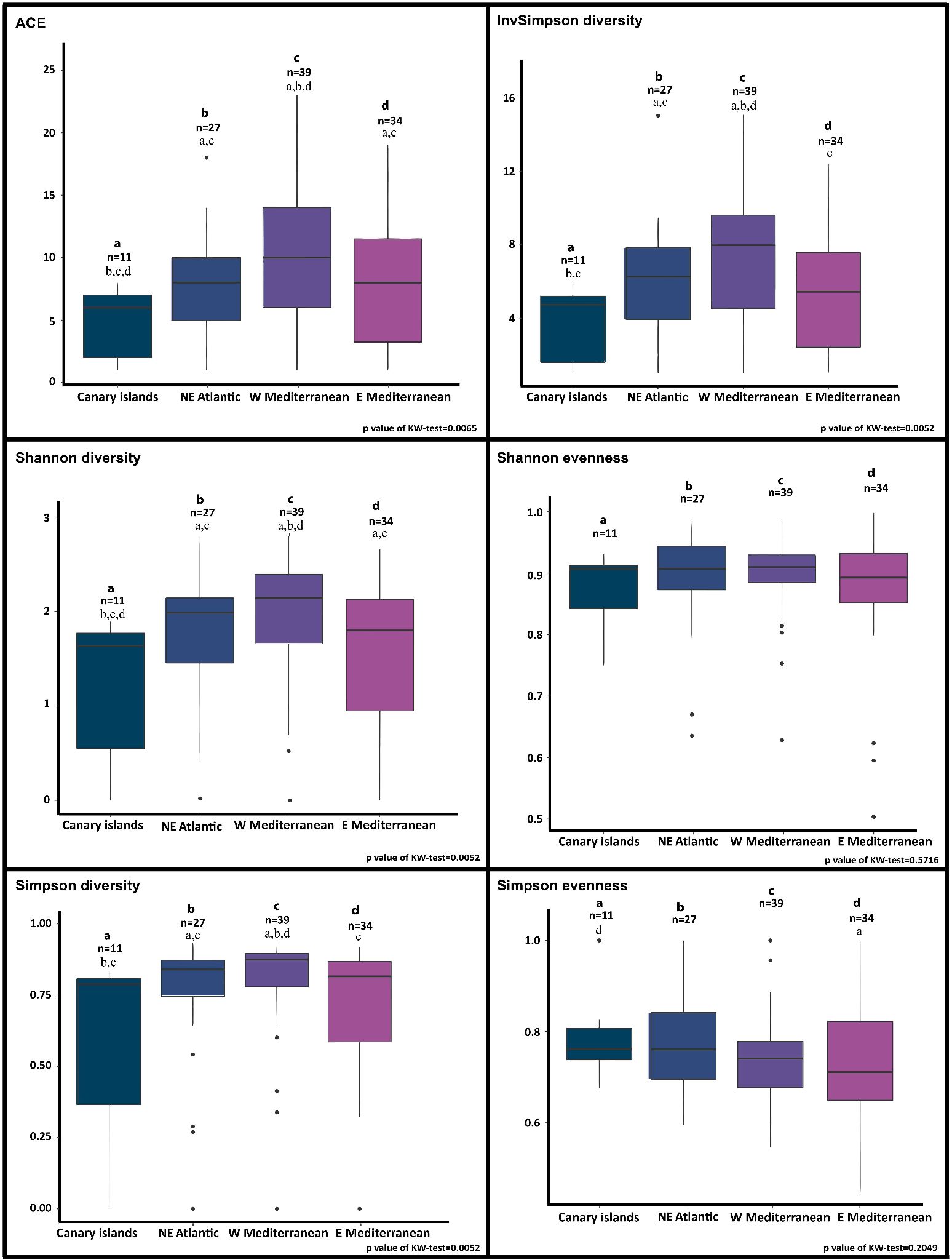
**

**Fig. S9:** Patterns of Alpha (boxplot) diversity of dicyemid communities in the Atlantic and Mediterranean regions analyzed in this study (letters below the sample numbers represent localities different from the selected one in a Kruskal–Wallis test).

**
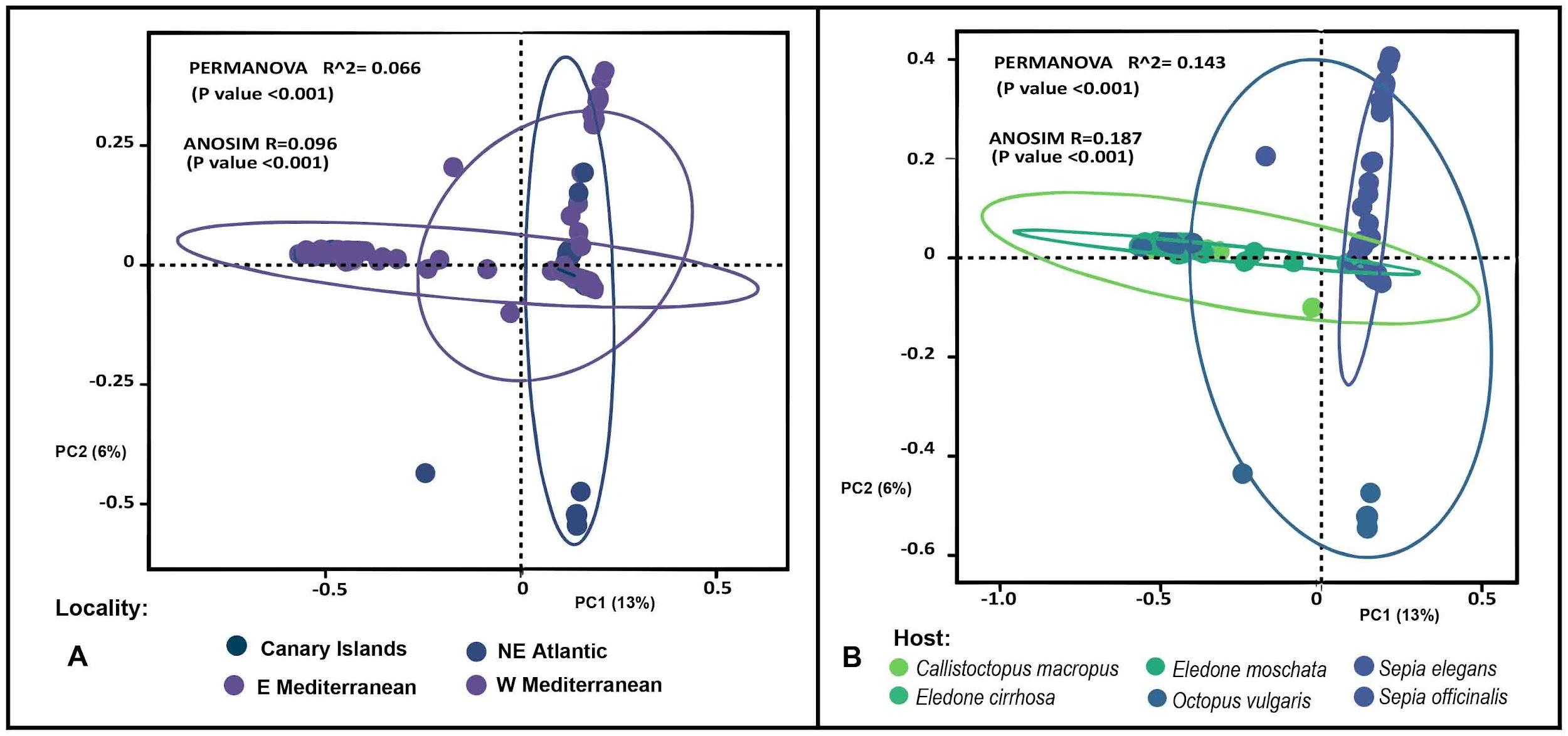
**

**Fig. S10:** Patterns of Beta (PCA) diversity of dicyemid communities in the Atlantic and Mediterranean regions: **A.** Diversities structured by the local regions analyzed in this study; **B.** Diversities structured by hosts occur in the Atlantic and Mediterranean regions.

**Literature:**

de Muinck EJ, Trosvik P, Gilfillan GD, Hov JR, Sundaram AY (2017) A novel ultra high-throughput 16S rRNA gene amplicon sequencing library preparation method for the Illumina HiSeq platform. Microbiome, 5(1). https://doi.org/10.1186/s40168-017-0279-1

Folmer O, Black M, Hoeh W, Lutz R, Vrijenhoek R (1994) DNA primers for amplification of mitochondrial cytochrome c oxidase subunit I from diverse metazoan invertebrates. Mol Mar Biol Biotechnol 3:294–299.

Hafner MS, Sudman PD, Villablanca FX, Spradling TA, Demastes JW, Nadler SA (1994) Disparate rates of molecular evolution in cospeciating hosts and parasites. Sci 265:1087–1090. https://doi.org/10.1126/science.8066445

**List of bioinformatic commands used in this study:**

# STEPS ON ITS AMPLICON SEQUENCING PROCESSING - DATA FROM NOVOGENE

### 1. Set up of the environment (to work from windows)

- Download and install SSHFS-Win

- Add shared folder using SSH (see below)

- Access via ssh using the following command

```bash

```

Files are located under this folder

```bash

/home/karol/data4karol/

```

Tutorial

https://docs.qiime2.org/2021.11/tutorials/moving-pictures/

https://docs.qiime2.org/2021.11/tutorials/overview/#let-s-get-oriented-flowcharts

### 2. Prepare the data for processing

#### 2.1 Download and decompress

Decompress and check the data folder structure of the data by Novogene. Files from Novogene will be like result_X_PROJECTNUMBER. Decompress it and it will contain several folders, with raw data, clean data and data analysis. The folder of interest will depend on us. For data processing, we will use data on 00.RawData folder.

#### 2.2 Create the manifest file

We will create a file called manifest.csv containing the paths to fastq files. The file should contain 3 main columns:

```bash

<SAMPLE NAME> <PATH TO R1> <PATH TO R2>

```

That is:

### 3. Process the data on QIIME2

Before doing anything else. We should activate the qiime2 conda environment:

```bash

conda activate qiime2-2021.11

```

#### 3.1 Create an artefict (single file) containing all DNA sequences

Please check the following to ensure file names are the same.

```bash

qiime tools import --type 'SampleData[PairedEndSequencesWithQuality]' --input-path manifest.csv --output-path soil4qiime.qza --input-format PairedEndFastqManifestPhred33V2

```

NOTES

- Only "manifest.csv" should be modified, according to the step 2.2 (manifest)

#### 3.2 Denoising and clustering into ASVs.

```bash

qiime dada2 denoise-paired --i-demultiplexed-seqs soil4qiime.qza --o-representative-sequences rep-seqs-dada2.qza --o-table table-dada2.qza --o-denoising-stats stats-dada2.qza --p-n-threads 0 --p-trunc-len-f 0 --p-trunc-len-r 0

```

#### 3.3 Taxonomy

```bash

qiime feature-classifier classify-sklearn --i-classifier /home/eduardo/tools/sh_qiime_release_10.05.2021/developer/unite-ver8-99-classifier-10.05.21.qza --i-reads rep-seqs-dada2.qza --o-classification taxonomy.qza --p-n-jobs 10

```

#### 3.4. Exclude unclassified sequences

```bash

qiime taxa filter-table --i-table table-dada2.qza --i-taxonomy taxonomy.qza --p-include p__ --o-filtered-table table-filtered.qza

```

#### 3.5 Filter representative seqs

```bash

qiime feature-table filter-seqs --i-table table-filtered.qza --i-data rep-seqs-dada2.qza --o-filtered-data rep-seqs-filtered.qza

```

### 4. Explore data and estimate diversity

A metadata file containing samples information should be prepared. Check this link for an example: https://data.qiime2.org/2021.4/tutorials/moving-pictures/sample_metadata.tsv

#### 4.1. Visualize data (i.e. nº of reads per sample, stats about representative DNA sequences...)

First, we have to create qzv files:

```bash

qiime metadata tabulate \

--m-input-file stats-dada2.qza \

--o-visualization stats-dada2.qzv

```

```bash

qiime feature-table tabulate-seqs \

--i-data rep-seqs-filtered.qza \

--o-visualization rep-seqs.qzv

```

```bash

qiime feature-table summarize \

--i-table table-filtered.qza \

--o-visualization table.qzv \

--m-sample-metadata-file metadata_inocas.txt

```

Second, to visualize them, we have to text:

```bash

qiime tools view stats-data2-qzv

qiime tools view rep-seqs.qzv

```

#### 4.2 Generate a tree for phylogenetic diversity analyses

We have to find a protocol to do it for fungi from ITS amplicon sequences...

#### 4.3. Alpha and beta diversity analysis

See https://docs.qiime2.org/2021.11/tutorials/moving-pictures/#alpha-and-beta-diversity-analysis

Importantly, the `--p-sampling-depth` needs to be carefully adjusted depending on the information on the table.qzv (see above).

```bash

qiime diversity core-metrics-phylogenetic \

--i-phylogeny rooted-tree.qza \

--i-table table-filtered.qza \

--p-sampling-depth 40000 \

--m-metadata-file metadata_inocas.txt \

--output-dir core-metrics-results

```

#### 4.4 Alpha rarefaction plotting

See https://docs.qiime2.org/2021.11/tutorials/moving-pictures/#alpha-rarefaction-plotting

Importantly, the `--p-max-pepth` needs to be carefully adjusted depending on the information on the table.qzv (see above).

```

qiime diversity alpha-rarefaction \

--i-table table-filtered.qza \

--i-phylogeny rooted-tree.qza \

--p-max-depth 45000 \

--m-metadata-file metadata_inocas.txt \

--o-visualization alpha-rarefaction.qzv

```

#### 4.5. Plot taxonomic information

See https://docs.qiime2.org/2021.11/tutorials/moving-pictures/#taxonomic-analysis

```bash

qiime metadata tabulate \

--m-input-file taxonomy.qza \

--o-visualization taxonomy.qzv

```

```bash

qiime taxa barplot \

--i-table table-filtered.qza \

--i-taxonomy taxonomy.qza \

--m-metadata-file metadata_inocas.txt \

--o-visualization taxa-bar-plots.qzv
